# Reduced temporal turnover in carabid communities enhances biomass stability in agricultural landscapes

**DOI:** 10.1111/1365-2656.70063

**Published:** 2025-05-29

**Authors:** Lucile Muneret, Audrey Alignier, Benoît Ricci, Alexandre Dosset, Roland Allart, Chantal Ducourtieux, Emilien Laurent, Stéphanie Aviron, Aude Vialatte, Sandrine Petit

**Affiliations:** ^1^ Agroécologie INRAE, Institut Agro, Université Bourgogne, Université Bourgogne Franche‐Comté INRAE, Institut Agro, Université Bourgogne Europe Dijon France; ^2^ Université Paris‐Saclay, AgroParisTech, INRAE, UMR Agronomie Palaiseau France; ^3^ UMR BAGAP, INRAE–Institut Agro Rennes Angers–ESA Rennes France; ^4^ LTSER Zone Atelier Armorique Rennes France; ^5^ ABSys, Université Montpellier, CIHEAM‐IAMM, CIRAD, INRAE, Institut Agro Montpellier France; ^6^ Université de Toulouse, INRAE, DYNAFOR Castanet‐Tolosan France

**Keywords:** agricultural practices, arthropod biomass, asynchrony, crop diversification, species richness, variability, β‐diversity, γ‐richness

## Abstract

1. While the temporal stability of plant communities has been well investigated, almost nothing is known about the inter‐annual stability of arthropod communities, especially those inhabiting highly disturbed habitats such as croplands. We investigated the effects of landscape, management and community drivers on the temporal carabid biomass stability and its mean in agricultural landscapes.
2. The dataset was composed of carabids collected in 57 arable agroecosystems from three areas in France excluding pastures and managed under organic, conservation or conventional farming. Data also included local‐ and landscape‐level management descriptors.
3. Through a piecewise structural equation modelling approach, we tested the effects of landscape‐ and field‐level management intensity and variability, richness and compositional carabid community metrics such as mean α‐richness, standard deviation α‐richness, β‐diversity and asynchrony on carabid biomass stability and mean value.
4. The temporal stability of the carabid biomass increased as temporal β‐diversity of the carabid community decreased and asynchrony among species increased. Furthermore, β‐diversity decreased and asynchrony increased with the community's mean α‐richness. At the landscape level, diversifying crop phenology and reducing the proportion of cropland appear to be the most efficient way to support carabid biomass stability.
5. The effect of α‐richness on carabid biomass stability tends to be modulated by species composition. However, this study underscores the need to investigate the relative effects of temporal β‐diversity and asynchrony on the stabilisation of ecosystem functioning in real ecosystems, as the reasons for their lack of covariance and similar effects remain unclear.

## Graphical abstract

The interannual stability of the carabid biomass in agricultural fields can be maximised by supporting the in‐field mean alpha‐richness that reduces their temporal beta‐diversity. Increasing crop phenology diversity at the landscape level also stabilises carabid biomass over time.

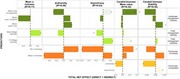

## Résumé

1. Bien que la stabilité temporelle des communautés végétales ait été bien étudiée, des trous de connaissances existent sur la stabilité interannuelle des communautés d'arthropodes, particulièrement en milieux cultivés. Nous avons étudié les effets des facteurs liés au paysage, à la gestion agricole et aux richesse et composition de la communauté de carabes sur la stabilité temporelle de la biomasse des carabes et sa moyenne dans les paysages agricoles.
2. Le jeu de données était composé de carabes collectés dans 57 agroécosystèmes arables de trois régions de France, à l'exclusion des pâturages, et gérés selon des pratiques biologiques, de conservation ou conventionnelles. Les données incluaient également des descripteurs de gestion agricole au niveau local et du paysage.
3. Grâce à une approche de modélisation par équations structurelles par morceaux, nous avons testé les effets de l'intensité et de la variabilité de la gestion agricole au niveau du paysage et des champs, de la richesse, et des métriques de composition de la communauté de carabes telles que la richesse α moyenne, l'écart‐type de la richesse α, la β‐diversité et l'asynchronie sur la stabilité et la moyenne de la biomasse des carabes.
4. La stabilité temporelle de la biomasse des carabes a augmenté à mesure que la β‐diversité temporelle de la communauté de carabes diminuait et que l'asynchronie entre les espèces augmentait. De plus, la β‐diversité a diminué et l'asynchronie a augmenté avec la richesse α moyenne de la communauté. Au niveau du paysage, la diversification de la phénologie des cultures et la réduction de la proportion de terres cultivées semblent être les moyens les plus efficaces pour soutenir la stabilité de la biomasse des carabes.
5. L'effet de la richesse α sur la stabilité de la biomasse des carabes tend à être modulé par la composition des espèces. Cependant, cette étude souligne la nécessité d'étudier les effets relatifs de la β‐diversité temporelle et de l'asynchronie sur la stabilisation du fonctionnement des écosystèmes dans des écosystèmes réels, car les raisons de leur manque de covariance et d'effets similaires restent floues.

## 1 INTRODUCTION

Temporal stability of biodiversity is crucial for the maintenance of ecosystem processes over time since plant and animal communities are involved in the key functions underlying many production and regulatory services (Hautier et al., 2015; Kleijn et al., 2015). Evidence shows that increasing community richness/diversity promotes the temporal stability of biomass production in both plant and animal communities (van der Plas, 2019). Various mechanisms have been hypothesised to explain the positive correlations between the diversity of a taxonomic or functional group and its community‐wide biomass stability (e.g. Conti et al., 2023; Sasaki & Lauenroth, 2011). First, local richness can stabilise a given function because demographic stochasticity increases the probability of having a species that provides the function (Doak et al., 1998; Loreau et al., 2021). Second, community composition can modulate the α‐richness‐stability relationship, as revealed by studies on plant communities (e.g. Craven et al., 2018). Locally, the co‐occurrence of species that are efficient for (for example a specialist species of a given prey), complementary or even stable at delivering a function is expected to increase the stability as well as the mean of the function (Grman et al., 2010; Hector et al., 2010; Yan et al., 2021). Additionally, species can be asynchronous, likely due to inter‐specific competition or environmental fluctuations over time, and this may lead to compensatory dynamics that should promote stability (Blüthgen et al., 2016; Valencia et al., 2020; Xu et al., 2021). This pattern can be even strengthened when communities are composed of functionally dissimilar species (Craven et al., 2018; Lepš et al., 2018; van Klink et al., 2019). Finally, community evenness is also expected to modulate the α‐richness‐stability relationship, although the direction of such an effect is ambiguous (Grman et al., 2010; Hillebrand et al., 2008; Isbell et al., 2009; Sasaki & Lauenroth, 2011). Furthermore, at broader spatial or temporal scales, β‐diversity is a widely used compositional metric, but its effect on the stability of ecosystem functioning has received little attention (Mori et al., 2018). Focusing on temporal β‐diversity (i.e. temporal turnover) should clarify to what extent a set of species is stable over time and whether species offset each other at providing a particular function. Moreover, the effect of β‐diversity at stabilising the community biomass should depend on the mechanisms linking α‐richness and β‐diversity (detailed below).

Beyond the local community‐scale mechanisms linking species richness to the stability of ecosystem functioning, the environmental context influences the stability of functioning (Grman et al., 2010). Increasing land use intensity has negative net effects on community stability by decreasing asynchrony or population stability (Blüthgen et al., 2016; Olivier et al., 2020). However, the literature is largely dominated by studies on plants, mostly in grasslands or forests (i.e. habitats less disturbed by anthropic activities than agroecosystems) (Xu et al., 2021). It is unknown whether mechanisms stabilising local ecosystem functioning for plants can be extrapolated to other communities or habitats. Specifically, temporal β‐diversity might play an overriding role in situations where environmental fluctuations and disturbances are important since dispersal strongly influences species assembly (van der Plas et al., 2023), like in arable agroecosystems. However, the relationship between β‐diversity and the stability of ecosystem functioning is expected to depend on the process underpinning the level of turnover of a community because β‐diversity might not respond linearly to increasing disturbance levels (Socolar et al., 2016). Particularly, at very low levels of disturbance, some rare native species may be lost but can be compensated by invasive species (referred to as subtractive and additive heterogenization, respectively), which might lead to a high β‐diversity and a high α‐richness, potentially associated with a high stability of functioning. At intermediate levels of disturbance, rare species may completely disappear (subtractive homogenization), while generalist species, including invasive ones, dominate the community (additive homogenization), potentially decreasing β‐diversity as well as α‐richness, reducing the stability of functioning. Finally, at very high levels of disturbance, when the total abundance of the community is very low, even generalist species may be lost, which might lead to high β‐diversity with very low α‐richness, which might reduce even more the stability of functioning.

Therefore, increasing β‐diversity through species loss or gain could have opposing effects (negative and positive, respectively) on the stability of ecosystem functioning.

The wide array of disturbances experienced by agricultural landscapes likely contributes to the idiosyncratic effect of β‐diversity on different communities (Alignier & Baudry, 2016; Baselga et al., 2015; Buhk et al., 2017; Clough et al., 2007). Conversely, very little is known about the temporal stability of the natural enemy community, mostly due to a dearth of long‐term data in agroecosystems (Petit et al., 2023). Natural enemies provide pest and weed control services in agroecosystems, notably carabids, a highly diversified family including phytophagous, omnivorous and carnivorous species. A few studies have shown that the composition of this community or the one‐shot level of pest control services can be affected by temporal variations of the environment such as interannual crop cover dynamics (Bertrand et al., 2016; Rusch et al., 2013; Schneider et al., 2015). There have also been some rare attempts to identify the management drivers of pest control intra‐annual variability in specific crops (Scheiner & Martin, 2020). However, nothing is known about the inter‐annual stability of the natural enemy community, while there is a pressing need to document both the mean and the stability of the delivery of ecosystem services to convince farmers to adopt biodiversity‐friendly practices.

**FIGURE 1:**
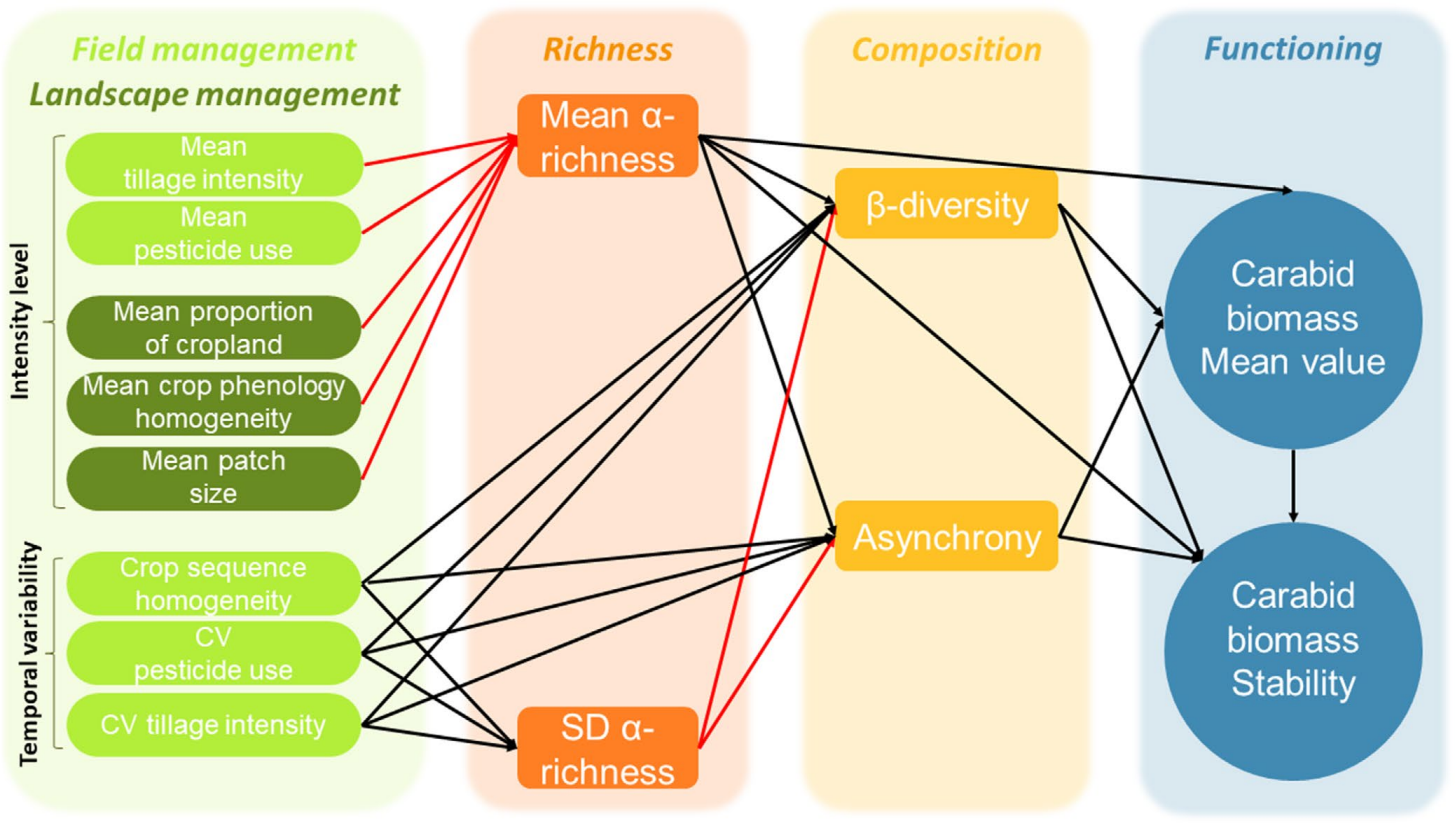
Hypotheses‐based conceptual framework linking field and landscape management, carabid community α‐richness, composition and carabid biomass mean value and stability (aggregated over the 4 years). Red and black arrows indicate expected negative and positive causal relationships respectively.

In this study, we examine the drivers of the interannual stability of carabid biomass for 4 years in 57 arable agroecosystems under contrasting farming management intensities. The temporal stability of the agrobiont carabid community has never been studied while the stability of carabid abundance observed in natural ecosystems showed no response to isolated disturbances like fire or topsoil removal (van Klink et al., 2019 but authors had a restricted dataset to analyse this). Also, it has been shown that extensively managed agroecosystems had a beneficial effect on carabid abundance and diversity (Caro et al., 2016). This can be exacerbated by an extensively managed landscape in some cases only (Caro et al., 2016; Petit et al., 2017). Community biomass is relevant when expressing production services for both primary and secondary productivity (Kempel et al., 2023; Xu et al., 2021). It also reflects energy passing from lower to higher trophic levels, and is hence highly relevant to studying links between trophic levels (Barnes et al., 2020; Borer et al., 2012) and therefore ecosystem functioning. Through a structural equation modelling approach, we tested the causal links between land use intensity, community richness, composition and both carabid biomass mean value and stability. First, we expected that the composition of the community, particularly asynchrony and β‐diversity modulate the effect of α‐richness on the carabid biomass stability (Figure 1). Specifically, increasing the in‐field mean α‐richness might lead to increasing both asynchrony and β‐diversity that in turn stabilise the carabid biomass. Second, we expected that the mean local‐ and landscape‐level management intensity decreases the carabid mean α‐richness. In a complementary way, we assumed that the variation of the local‐ and landscape‐level management intensity increases the variation of in‐field α‐richness that in turn decreases both asynchrony and β‐diversity linked to the loss of species some years (in situations of intensive management) as well as the stability of the carabid biomass.

## 2 MATERIALS AND METHODS

### 2.1 Study site

Fifty‐seven arable agroecosystems were surveyed over 4 years (2014, 2015, 2016 and 2018) in three French areas (near Dijon 47.32 N, 5.04 E; Rennes 48.12 N, −1.68 E; Toulouse 43.60, 1.44; SEBIOPAG Network; Figure S1; Petit et al., 2021). They were distributed along a cropland proportion gradient in the 1 km^2^ surrounding landscape (from 22% to 95%, Figure S2) and included a wide diversity of farming systems including organic farming, conservation agriculture and conventional agriculture including contrasting tillage strategies (*N* = 13,11 and 33 respectively; Muneret et al., 2023). Farmers were interviewed each year to collect information about their practices (i.e. tillage strategy, pesticide use intensity and crop diversity).

### 2.2 Experimental design

Carabids were sampled using four pitfall traps (10 cm deep × 8 cm diameter) placed along two parallel transects that were 10 m apart and running perpendicular to the field edge. Two traps were placed at 10 m and two were placed at 50 m from the field edge. Traps were opened for 5 days twice a year, at 1100 and 1500 degree‐days for winter crops (i.e. crop vegetative growth period and ear or pod maturation stage, respectively). The number of degree‐days was the sum of daily mean temperatures from the 1st of January that were measured on each meteorological site using the Climatik database (https://agroclim.inrae.fr). For summer crops, the former sampling took place at 1500 degree‐days and the latter was performed after about the same interval of time between the two dates of winter crop samplings (Appendix S1). Specimens were identified at the species level except for some rare individuals (references used reported in Appendix S1). The ‘annual carabid community’ was the sum of individuals caught in the four traps at the two sampling dates in a field. Given the measurements taken in the field, our study did not require any special permission or ethical approval.

### 2.3 Data preparation

Field management intensity was described by five metrics (Table S1). First, we calculated over the 4‐year crop sequence, the mean annual value and the ‘temporal’ Coefficient of Variation (“CV”) of tillage intensity (number of tillage operations between the harvest of crop year *n* − 1 and the harvest of crop year *n*) and pesticide use intensity (treatment frequency index) during this same time (Muneret et al., 2023). The treatment frequency index reflects the intensity of pesticide application relative to the recommended dose but does not consider the toxicity of active substances (see formula in Table S1). A fifth metric describing the crop phenology homogeneity along the crop sequence (i.e. the Shannon index multiplied by minus one) was calculated for each field by classification of crops into three broad types; winter or summer crops or rotational temporary grasslands (Table S1).

Landscape management intensity was estimated by four metrics; the mean annual proportion of cropland, the mean patch size area and the mean as well as the CV of the crop phenology homogeneity (using an annual Shannon index based on winter crops, summer crops and rotational temporary grasslands; Table S1). However, as the mean and CV of the crop phenology homogeneity were highly correlated (Pearson correlation = 0.86, Figure S3), we only kept the mean crop phenology homogeneity for further analyses. These metrics were calculated in a circular buffer of 1 km^2^ area around the 57 fields each year using the ‘alm’ package (Allart et al., 2021).

Carabid biomass was estimated as *Body mass = 0.0237 × Body size* ^*2.7054*^ (Barnes et al., 2014). The body size of each carabid species was extracted from the literature (Appendix S1) and total carabid biomass per field and per year was estimated by multiplying the number of specimens by the mean body mass of each species. We then calculated the carabid biomass mean value (μ) and stability (μ/σ) per field over the 4 years. For sensitivity analyses, carabid biomass stability was also calculated as CV^−1^ (σ/μ^−1^; Table S1).

We calculated three community richness metrics: the mean ɑ‐richness, its standard deviation ‘SD ɑ‐richness’ and the species pool ‘ɣ‐richness’ (i.e. the total number of species detected in a given field). We also calculated two compositional metrics: ꞵ‐diversity (the Sorensen‐based multiple‐site dissimilarity metric (Baselga et al., 2023), function ‘beta.multi’, betapart package; Baselga et al., 2023), which is the temporal turnover of the community for each field here, Asynchrony (function ‘synchrony’ in the codyn package, multiplied by −1, Gross et al., 2014; Hallett et al., 2020). Synchrony measures the averaged correlation between individual species biomass and the biomass of all the other species of the community over time (Table S1).

### 2.4 Conceptual framework and statistical analyses

To identify the drivers of the carabid biomass mean value and stability, we used a piecewise structural equation modelling (SEM) (Lefcheck, 2016) based on 57 observations. First, we developed a hypotheses‐based conceptual framework including expected causal links between (i) field and landscape‐level management intensity and variability, (ii) carabid community α‐richness and composition, and (iii) carabid biomass mean value and stability that reflect the level of agroecosystem functioning (Figure 1; references and details about the hypotheses are in the Table S2).

All the variables were standardised before modelling and the absence of collinearity among predictors was checked (Figures S3 and S4). In SEM, the hypotheses from the conceptual framework were translated into six linear models and a potential co‐variation between mean and SD α‐richness was included (Appendix S2). All the models included an offset to consider the variability of the sampling effort due to in‐field trap destruction, namely the total number of traps used to collect carabids (log‐transformed; from 22 to 32). All missing paths detected were added to the hypothetical SEM. Then, we applied a multimodal inference with delta Akaike information criterion (AIC) = <2 for each model obtained using the ‘dredge’ function (Burnham et al., 2011) to simplify the models in the SEM. We then developed an updated version of SEM based only on the significant relationships detected using the dredge function. This step enhances AIC because it removes all non‐significant hypothetical relationships. To further improve SEM, missing paths among composition metrics were included as correlations in SEM. Finally, we checked VIF and residuals of the final models in the SEM and applied a D‐test for model validation. Direct, indirect and total effects were calculated using the ‘semEff’ package (Murphy, 2022). All analyses were performed using R (v4.3.1, R Core Team, 2021).

We performed sensitivity analyses to evaluate the influence of richness and stability metrics using three other versions of the SEM. To examine the effect of the ɣ‐richness instead of mean ɑ‐richness on the carabid biomass mean value and stability, we applied the same procedure but mean ɑ‐richness was replaced by ɣ‐richness. These variables were too positively correlated to be included in the same SEM (*R* ^2^ = 0.86). To ensure that our results were not dependent on the choice of stability metric, we ran another two versions of SEM using an alternative metric of the carabid biomass stability: the opposite of CV (σ/μ)^−1^ as described in Blüthgen et al. (2016). The third version of the SEM included ɣ‐richness instead of mean ɑ‐richness and the opposite of CV (σ/μ)^−1^ instead of μ/σ as the carabid biomass stability metric. We tested for the absence of spatial autocorrelation in the residuals of all models included in the piecewise structural equation models using Moran's tests.

## 3 RESULTS

**FIGURE 2:**
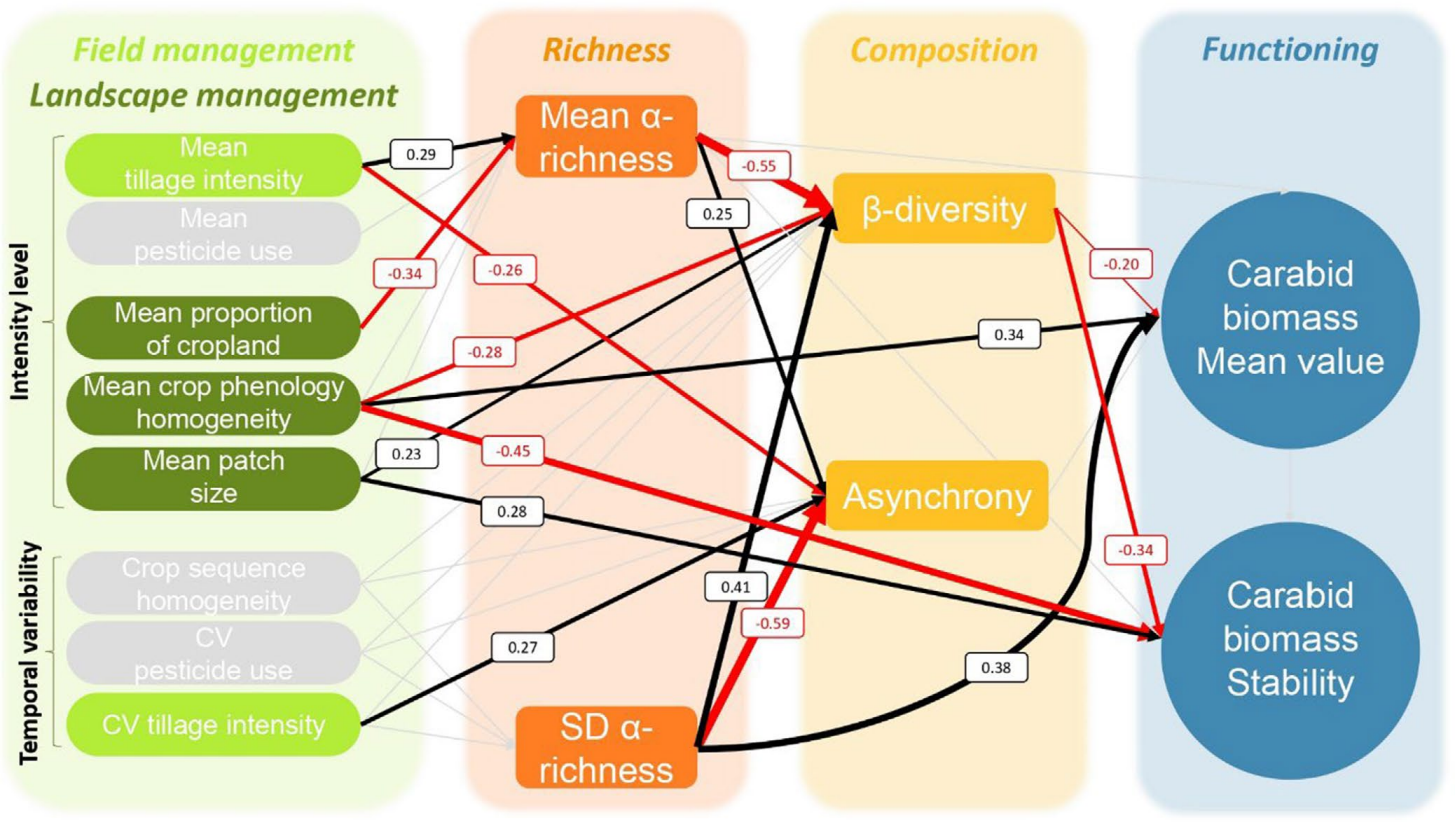
Direct effects of the drivers of the carabid biomass mean value and stability. Black and red arrows indicate positive and negative effects, respectively. Associated values and arrow thickness report estimates from the final SEM (Table 1). Marginal effects of such relationships are presented in Appendix S3.

**FIGURE 3:**
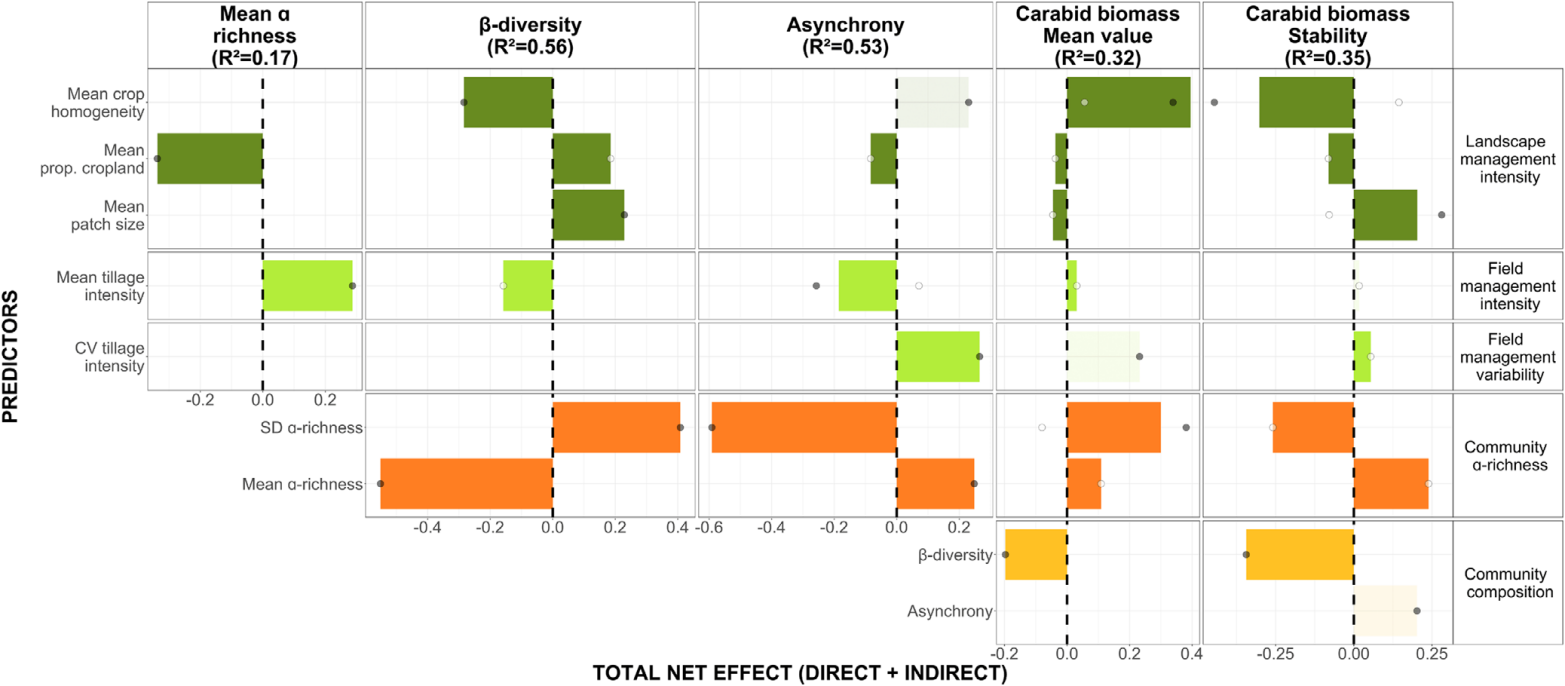
Total (i.e. direct + indirect) effects of the drivers of the carabid biomass mean value and stability. Values were estimated using the semEff package (Murphy, 2022). Response variables appear at the top of the diagram. Shaded bars represent retained but insignificant effects (Table 1). Dark grey and white dots are direct and indirect effects. Only metrics retained in the final SEM are shown.

A total of 15,968 carabids (113 taxa; Table S3) were collected in the 57 fields between 2014 and 2018. There were on average between 37 and 2185 specimens per field over the 4 years. The carabid biomass mean value was highly variable in space and time; the mean biomass ranged from 129 mg to 6467 mg and its temporal stability varied from 0.67 to 3.36. Asynchrony and ꞵ‐diversity varied from −0.77 to 0.20 and from 0.50 to 0.94 respectively. The final SEM represented the data well, no missing paths were detected (*χ* ^2^ = 9.5; *p* = 0.97, 20 degrees of freedom; Fischer's *C* = 20.62; *p* = 0.99; 40 degrees of freedom; AIC = 711). The carabid biomass mean value and stability were decently explained by SEM (*R* ^2^ = 0.32 and *R* ^2^ = 0.35, respectively; Table 1, Figure 2). We detected no correlation between carabid biomass mean value and stability (Table 1). Significant direct effects only are presented in Figure 2 and in Appendix S3 and total net effects are presented in Figure 3.

**TABLE 1:** Direct and indirect effects of the predictors based on the final version of the structural equation model (SEM).

Response	Path	Predictor	Est.	Bias	SE	LCI	UCI
Mean	Direct	**Mean prop. cropland**	**−0.337**	**0.009**	**0.108**	**−0.540**	**−0.134**
α‐richness		**Mean tillage int**.	**0.287**	**−0.003**	**0.105**	**0.028**	**0.477**
(*R* ^2^ = 0.17)	Total	**Mean prop. cropland**	**−0.337**	**0.009**	**0.108**	**−0.540**	**−0.134**
		**Mean tillage int**.	**0.287**	**−0.003**	**0.105**	**0.028**	**0.477**
β‐diversity	Direct	**Mean patch size**	**0.229**	**−0.011**	**0.098**	**0.025**	**0.422**
(*R* ^2^ = 0.56)		**Mean crop pheno. homog**.	**−0.284**	**0.010**	**0.097**	**−0.494**	**−0.114**
		**SD α richness**	**0.408**	**−0.008**	**0.084**	**0.221**	**0.552**
		**Mean α richness**	**−0.551**	**0.032**	**0.105**	**−0.718**	**−0.386**
	Indirect	**Mean prop. cropland**	**0.185**	**−0.014**	**0.066**	**0.065**	**0.331**
		**Mean tillage int**.	**−0.158**	**0.015**	**0.055**	**−0.260**	**−0.077**
	Total	**Mean prop. cropland**	**0.185**	**−0.014**	**0.066**	**0.065**	**0.331**
		**Mean tillage int**.	**−0.158**	**0.015**	**0.055**	**−0.260**	**−0.077**
		**Mean patch size**	**0.229**	**−0.011**	**0.098**	**0.025**	**0.422**
		**Mean crop pheno. homog**.	**−0.284**	**0.010**	**0.097**	**−0.494**	**−0.114**
		**SD α richness**	**0.408**	**−0.008**	**0.084**	**0.221**	**0.552**
		**Mean α richness**	**−0.551**	**0.032**	**0.105**	**−0.718**	**−0.386**
	Med.	Mean α richness	0.027	0.001	0.079	−0.116	0.217
Asynchrony	Direct	**Mean tillage int**.	**−0.257**	**0.014**	**0.084**	**−0.473**	**−0.129**
(*R* ^2^ = 0.53)		Mean crop pheno. homog.	0.230	0.006	0.093	−0.020	0.375
		**SD α richness**	**−0.591**	**0.026**	**0.117**	**−0.818**	**−0.414**
		**CV tillage int**.	**0.265**	**0.001**	**0.086**	**0.066**	**0.431**
		**Mean α richness**	**0.248**	**0.002**	**0.108**	**0.043**	**0.491**
	Indirect	**Mean prop. cropland**	**−0.083**	**0.003**	**0.044**	**−0.202**	**−0.014**
		**Mean tillage int**.	**0.071**	**−0.003**	**0.039**	**0.014**	**0.219**
	Total	**Mean prop. cropland**	**−0.083**	**0.003**	**0.044**	**−0.202**	**−0.014**
		**Mean tillage int**.	**−0.185**	**0.011**	**0.072**	**−0.336**	**−0.032**
		Mean crop pheno. homog.	0.230	0.006	0.093	−0.020	0.375
		**SD α richness**	**−0.591**	**0.026**	**0.117**	**−0.818**	**−0.414**
		**CV tillage int**.	**0.265**	**0.001**	**0.086**	**0.066**	**0.431**
		**Mean α richness**	**0.248**	**0.002**	**0.108**	**0.043**	**0.491**
	Med.	Mean α richness	−0.012	0.000	0.048	−0.132	0.082
Carabid biomass	Direct	**Mean crop pheno. homog**.	**0.339**	**−0.024**	**0.135**	**0.054**	**0.607**
		**SD α richness**	**0.381**	**−0.010**	**0.104**	**0.142**	**0.593**
Mean value		CV tillage int.	0.232	−0.025	0.116	−0.021	0.409
(*R* ^2^ = 0.32)		**β‐diversity**	**−0.197**	**0.003**	**0.077**	**−0.429**	**−0.086**
	Indirect	**Mean prop. cropland**	**−0.037**	**0.002**	**0.022**	**−0.097**	**−0.014**
		**Mean tillage int**.	**0.031**	**−0.004**	**0.016**	**0.014**	**0.079**
		**Mean patch size**	**−0.045**	**0.001**	**0.028**	**−0.174**	**−0.002**
		**Mean crop pheno. homog**.	**0.056**	**−0.003**	**0.029**	**0.019**	**0.189**
		**SD α richness**	**−0.080**	**0.004**	**0.036**	**−0.234**	**−0.040**
		**Mean α richness**	**0.109**	**−0.005**	**0.052**	**0.045**	**0.308**
	Total	**Mean prop. cropland**	**−0.037**	**0.002**	**0.022**	**−0.097**	**−0.014**
		**Mean tillage int**.	**0.031**	**−0.004**	**0.016**	**0.014**	**0.079**
		**Mean patch size**	**−0.045**	**0.001**	**0.028**	**−0.174**	**−0.002**
		**Mean crop pheno. homog**.	**0.395**	**−0.027**	**0.129**	**0.144**	**0.637**
		**SD α richness**	**0.300**	**−0.007**	**0.100**	**0.096**	**0.508**
		CV tillage int.	0.232	−0.025	0.116	−0.021	0.409
		**Mean α richness**	**0.109**	**−0.005**	**0.052**	**0.045**	**0.308**
		**β‐diversity**	**−0.197**	**0.003**	**0.077**	**−0.429**	**−0.086**
	Med.	Mean α richness	−0.005	−0.002	0.018	−0.071	0.018
		β‐diversity	0.034	−0.005	0.033	−0.021	0.150
Carabid biomass	Direct	**Mean patch size**	**0.281**	**−0.002**	**0.088**	**0.129**	**0.475**
		**Mean crop pheno. homog**.	**−0.446**	**0.005**	**0.077**	**−0.597**	**−0.298**
Stability		**β‐diversity**	**−0.344**	**0.010**	**0.119**	**−0.689**	**−0.129**
(*R* ^2^ = 0.35)		Asynchrony	0.202	−0.022	0.126	−0.009	0.589
	Indirect	**Mean prop. Cropland**	**−0.081**	**0.008**	**0.032**	**−0.150**	**−0.033**
		Mean tillage int.	0.017	0.002	0.041	−0.068	0.110
		**Mean patch size**	**−0.079**	**0.006**	**0.042**	**−0.208**	**−0.007**
		**Mean crop pheno. homog**.	**0.144**	**−0.012**	**0.052**	**0.077**	**0.268**
		**SD α richness**	**−0.259**	**0.022**	**0.066**	**−0.432**	**−0.166**
		**CV tillage int**.	**0.054**	**−0.009**	**0.030**	**0.014**	**0.139**
		**Mean α richness**	**0.239**	**−0.021**	**0.064**	**0.138**	**0.371**
	Total	**Mean prop. Cropland**	**−0.081**	**0.008**	**0.032**	**−0.150**	**−0.033**
		Mean tillage int.	0.017	0.002	0.041	−0.068	0.110
		**Mean patch size**	**0.203**	**0.004**	**0.097**	**0.045**	**0.439**
		**Mean crop pheno. homog**.	**−0.302**	**−0.007**	**0.091**	**−0.505**	**−0.104**
		**SD α richness**	**−0.259**	**0.022**	**0.066**	**−0.432**	**−0.166**
		**CV tillage int**.	**0.054**	**−0.009**	**0.030**	**0.014**	**0.139**
		**Mean α richness**	**0.239**	**−0.021**	**0.064**	**0.138**	**0.371**
		**β‐diversity**	**−0.344**	**0.010**	**0.119**	**−0.689**	**−0.129**
		Asynchrony	0.202	−0.022	0.126	−0.009	0.589
	Med.	Mean α richness	−0.012	0.001	0.033	−0.107	0.058
		β‐diversity	0.059	−0.007	0.056	−0.036	0.221
		Asynchrony	−0.023	0.003	0.054	−0.230	0.041

*Note*: Values were calculated using the ‘semEff’ package (Murphy, 2022). Bold text highlights significant relationships. In the ‘Path’ column, ‘Total’ means the sum of direct and indirect effects.

Abbreviations: Est, estimate; LCI, lower confidence interval; Med., mediators; SE, standard error; UCI, upper confidence interval.

### 3.1 Effect of community α‐richness and composition on the carabid biomass stability and mean value

Carabid biomass stability was indirectly influenced by α‐richness (Table 1; Figures 2 and 3; orange and yellow bars in Figure 3); it was positively influenced by mean α‐richness and negatively by SD α‐richness. Similarly, the carabid biomass mean value was positively correlated with mean α‐richness and SD α‐richness (Figures 2 and 3 and Appendix S3). Contrary to our expectations, β‐diversity was directly and negatively correlated with both carabid biomass stability and its mean value (Figures 2 and 3 and Appendix S3) whereas asynchrony was not a significant driver of carabid biomass stability and mean value but tended to increase stability (Figure 3, but sensitivity analyses revealed a significant effect of asynchrony on carabid biomass stability).

We also detected a dual effect of α‐richness on β‐diversity comprising a negative effect of mean α‐richness and a positive effect of SD α‐richness (Figures 2 and 3 and Appendix S3). The negative relationship between mean α‐richness and β‐diversity was even more pronounced for fields with low α‐richness (Figure S5). Conversely, asynchrony responded negatively to SD α‐richness and positively to mean α‐richness. We detected no relationship between β‐diversity and asynchrony (Figure S6).

### 3.2 Effect of field and landscape‐level management intensity and variability on carabid biomass stability and mean value

Landscape‐scale management had a much more important influence on carabid biomass stability and mean value than field‐scale management (Table 1; Figures 2 and 3; light and dark green bars in the Figure 3).

The most important abiotic driver of carabid biomass was mean crop homogeneity with an opposite effect on stability and mean value. Mean crop homogeneity reduced carabid biomass stability while it increased its mean value. The negative effect of mean crop homogeneity on carabid biomass stability was driven by an indirect effect on reducing β‐diversity, but it was compensated by a much stronger direct negative effect. The carabid biomass mean value responded positively to mean crop homogeneity via decreasing β‐diversity and by a strong direct positive effect (Figures 2 and 3 and Appendix S3). As a second abiotic driver, mean patch size increased carabid biomass stability while it slightly decreased its mean value. Additionally, mean patch size increased β‐diversity. Moreover, the mean proportion of cropland decreased carabid biomass stability and mean value, asynchrony, α‐richness and increased β‐diversity. Finally, at the field‐scale level, CV tillage intensity slightly enhanced carabid biomass stability and mean tillage intensity increased carabid biomass mean value.

### 3.3 Sensitivity of the analysis to richness and stability metrics

Sensitivity analyses based on ɣ‐richness instead of mean α‐richness and/or CV‐1 carabid biomass as a stability metric in three piecewise structural equation models confirmed the vast majority of our results (Appendix S4). However, the main difference arose from the effect of field‐scale management intensity, that is mean pesticide use and mean tillage intensity, which increased asynchrony among species, ultimately leading to increased carabid biomass stability. In the main version presented here, only mean tillage intensity had a weak negative effect on asynchrony and no effect on carabid biomass stability. Additionally, asynchrony had a positive effect on carabid biomass stability in all three alternative piecewise structural equation models, while reducing β‐diversity to stabilise carabid biomass was only significant when carabid biomass stability was calculated as (μ/σ), not when it was calculated as CV^−1^. No spatial autocorrelation was detected in any of the models using Moran's tests (Table S4).

## 4 DISCUSSION

We investigated the biotic and abiotic drivers of carabid biomass stability and mean values using a 4‐year temporal series in 57 agroecosystems. Carabid biomass stability and mean values were higher in fields with high mean α‐richness, lower temporal turnover of species (i.e. low β‐diversity, which is preferable for stabilising and maximising carabid biomass), and high asynchrony among species (the latter observation comes from sensitivity analyses in the Appendix S4). Sensitivity analyses revealed that the relationship between compositional metrics (β‐diversity and asynchrony) and carabid biomass stability depended on the type of stability descriptors used, highlighting the necessity of performing such analyses systematically. Regarding abiotic drivers, reducing the proportion of cropland and decreasing crop phenology homogeneity appear to be the most important factors for stabilising carabid biomass. However, the latter effect may negatively impact mean carabid biomass value.

The positive effect of α‐richness on carabid biomass mean value and stability aligns with the large body of literature demonstrating the positive correlation between α‐richness and functioning (Cardinale et al., 2012). The main finding here is that the relationship between richness and carabid biomass is also correlated with decreasing β‐diversity, the temporal turnover of the community. β‐diversity tends to modulate the effect of α‐richness on carabid biomass stability. In this context, increasing β‐diversity corresponds to a ‘subtractive heterogeneization’ (not an additive one) of the community as introduced by Socolar et al. (2016). This means that species are lost as β‐diversity increases. It happens when the richness of the community is very low. This statement was confirmed when we determined the correlations between α‐richness and β‐diversity for poorer communities (Figure S5); the relationship was even more negative. This shows that the community is so poor in some fields that almost 100% of it is renewed year‐to‐year. The fact that increasing temporal turnover of the community destabilises carabid biomass suggests that carabid species varied greatly in term of abundance and body mass according to years and therefore, they do not offset each other to maintain a certain level of biomass at the community level. It is therefore likely that it has huge consequences for the stability of pest and weed control. This also suggests that to stabilise the level of functioning, it might be preferable to adopt measures that target species either having the highest probabilities to remain in (or in close vicinity of) fields, or widespread and regionally abundant species.

The sensitivity analysis revealed that asynchrony tends to mediate the effect of richness on stability, as previously demonstrated in animal communities (Catano et al., 2020; Olivier et al., 2020). However, the fact that this effect is not supported by all versions of the PSEM suggests that, in agricultural landscapes, carabid species assembly might be governed by a ‘trait syndrome’ favouring small and opportunistic species (Gámez‐Virués et al., 2015; Lepš et al., 2018). Such potential community homogenization might limit its ability to cope with habitat variations, thereby reducing its capacity to maintain stable biomass and even ecosystem functions. Nevertheless, we robustly found that asynchrony responded positively to variation in in‐field tillage intensity, suggesting that a diversity of tillage intensities in the landscape could enhance the diversity of carabid life strategies (Muneret et al., 2023).

Another surprising result is the absence of a positive correlation between β‐diversity and asynchrony. We expected a positive co‐variation since both represent changes in community composition over time. However, the observed relationships between these two metrics and carabid biomass stability lead to inconsistent conclusions. When examining the link between β‐diversity and carabid biomass stability, we can conclude that carabid species do not compensate for each other to maintain a stable biomass. In contrast, when considering the link between asynchrony and carabid biomass stability, we reach the opposite conclusion. We did not find any studies investigating the relationship between these two concepts, but we suggest that exploring why they do not co‐vary could be highly interesting.

A striking result from our study is the overriding role of crop phenology homogeneity at the landscape scale, which has a positive effect on mean carabid biomass but a negative effect on its stability. Similarly, the mean proportion of cropland reduces both the mean carabid biomass and its stability. Landscape‐level management was a more important factor than field‐scale management, as mean tillage intensity and pesticide use intensity only slightly increased carabid biomass stability in the sensitivity analysis. Land‐use intensity has already been identified as a factor destabilising bat, bird, and butterfly communities in grasslands (Blüthgen et al., 2016; Olivier et al., 2020), but its relationship with carabid stability remains equivocal (Mei et al., 2023; Winqvist et al., 2011). Previous research has also shown that crop diversification at the landscape level can benefit multitrophic biodiversity and pest control services (Bosem Baillod et al., 2017; Redlich et al., 2021; Sirami et al., 2019), though its effect on stability has not been measured. To our knowledge, the effect of landscape or local management on the biomass of natural enemies is rarely documented (but see Bertrand et al., 2016). However, further research on community biomass stability is crucial, as these organisms provide key regulatory services in response to interannual variations in land use (Kempel et al., 2023).

We considered carabid biomass even though it is not commonly used in related studies and can be questioned. We argue that this metric is relevant because it can be interpreted as a proxy of pressure exerted on lower trophic levels by carabids, and a resource for higher trophic‐level communities such as shrews or birds. However, this assertion becomes even more defensible when considering a functionally diverse community of natural enemies, which suggests complementarity among species (Greenop et al., 2018). Moreover, in bioenergetics‐based studies, community or species biomass combined with diet preferences can be employed as a proxy for pest control services (Barnes et al., 2020). The difficulty here is that carabid species are highly opportunistic (Frei et al., 2019; Roubinet et al., 2018), but we could develop this approach through creating pest‐specific natural enemy pressure metrics based on natural enemy biomass and diet.

To conclude, the mean value and stability of carabid biomass can be enhanced by increasing their α‐ or ɣ‐richness, which tends to reduce the temporal turnover of the community (i.e. β‐diversity) while simultaneously increasing asynchrony. It is likely that the level of disturbances experienced by agricultural fields leads to trait syndromes that prevent asynchrony among species in poorer fields. This, coupled with recurrent annual field colonisation, results in subtractive heterogenization and low mean carabid biomass stability and value. Among local and landscape‐level factors, reducing the proportion of cropland and diversifying crop phenology at the landscape level appear to be the most effective ways to support carabid biomass stability.

## AUTHOR CONTRIBUTIONS

The study was conceived by Lucile Muneret and Sandrine Petit with inputs from Alexandre Dosset, Audrey Alignier, Benoît Ricci, Stéphanie Aviron and Aude Vialatte. Data were collected and managed by Roland Allart, Chantal Ducourtieux, and Emilien Laurent. Data were analysed by Lucile Muneret with contributions from Roland Allart, Alexandre Dosset, Benoît Ricci and advice from Sandrine Petit, Audrey Alignier, Stéphanie Aviron and Aude Vialatte. Sandrine Petit oversaw the project's funding; Lucile Muneret wrote the first draft and all authors contributed significantly to the final version.

## CONFLICT OF INTEREST STATEMENT

None to declare.

## Supporting information


**Table S1.** Description of all the variables used in the study.
**Table S2.** Hypotheses supporting the conceptual framework.
**Table S3.** Number of specimens for each species sampled in each area.
**Table S4.** Results of the Moran's tests showing there is no spatial autocorrelation in the final models included in the piecewise structural equation models.
**Figure S1.** Map of the 57 arable agroecosystems of the study.
**Figure S2.** Correlations between abiotic variables (“D”, “R” and “T” mean Dijon, Rennes and Toulouse area respectively).
**Figure S3.** Pearson correlations between the scaled abiotic variables.
**Figure S4.** Pearson correlations between the scaled biotic variables (richness, composition, functioning descriptors).
**Figure S5.** Relationships between mean α‐richness and β‐diversity for the poorer and richer fields (i.e. negative and positive values for mean α‐richness respectively after scaling).
**Figure S6.** Co‐variations between (i) mean and SD α‐richness and (ii) β‐diversity and Asynchrony.
**Appendix S1.** Sampling dates for the carabid community and references used for determining and assessing the body length of the carabid species.
**Appendix S2.** Set of models included the initial SEM presented in the main text.
**Appendix S3.** Relationships between mean α‐richness and (A) mean proportion of cropland, (B) Mean tillage intensity.
**Appendix S4.** The three alternative versions of the SEM. In each of the three sections, the figures and tables are presented.

## Data Availability

Data used to run the models are available at Data.gouv.fr: Muneret, Lucile, 2023, “Données de réplication pour: Species richness enhances the stability of arthropod biomass by decreasing their temporal turnover in agricultural landscapes”, https://doi.org/10.57745/GYKF3F, Recherche Data Gouv, V1. Scripts are available upon request.
