# Supplementary material for: Reduced temporal turnover in carabid communities enhances biomass stability in agricultural landscapes
Source: J Anim Ecol. 2025 May 29;94(7):1410–21. doi: 10.1111/1365-2656.70063 (PMC12214439; doi:10.1111/1365-2656.70063)
Supplement: Supplementary file 1 — Table S1. Description of all the variables used in the study. Table S2. Hypotheses supporting the conceptual framework. Table S3. Number of specimens for each species sampled in each area. Table S4. Results of the Moran's tests showing there is no spatial autocorrelation in the final models included in the piecewise structural equation models. Figure S1. Map of the 57 arable agroecosystems of the study. Figure S2. Correlations between abiotic variables (“D”, “R” and “T” mean Dijon, Rennes and Toulouse area respectively). Figure S3. Pearson correlations between the scaled abiotic variables. Figure S4. Pearson correlations between the scaled biotic variables (richness, composition, functioning descriptors). Figure S5. Relationships between mean α‐richness and β‐diversity for the poorer and richer fields (i.e. negative and positive values for mean α‐richness respectively after scaling). Figure S6. Co‐variations between (i) mean and SD α‐richness and (ii) β‐diversity and Asynchrony. Appendix S1. Sampling dates for the carabid community and references used for determining and assessing the body length of the carabid species. Appendix S2. Set of models included the initial SEM presented in the main text. Appendix S3. Relationships between mean α‐richness and (A) mean proportion of cropland, (B) Mean tillage intensity. Appendix S4. The three alternative versions of the SEM. In each of the three sections, the figures and tables are presented. [file JANE-94-1410-s001.docx]

**Supplementary material for: Reduced temporal turnover in carabid communities enhances biomass stability in agricultural landscapes**

**Contents**

Table S1, S2, S3, S4: pages 2−11

Figures S1-S6: pages 12-16

Appendix S1: pages 17-19

Appendix S2: page 20-21

Appendix S3: pages 22-24

Appendix S4: pages 25-33

**Table S1. Description of all the variables used in the study.** Abiotic (field and landscape level descriptors), biotic (community richness and composition descriptors) and functioning (i.e. carabid biomass) variables are described here.

| **Variable type** | **Descriptor of** | **Variable name** | **Definition** |
| --- | --- | --- | --- |
| Abiotic | Field | **Mean pesticide use** | Mean based on the 4-year crop sequence of the treatment frequency index* based on all types of pesticides (herbicides, fungicides, insecticides) applied between harvest n-1 and harvest n. |
|  |  | **CV pesticide use** | Coefficient of variation (CV) based on the 4-year crop sequence of the treatment frequency index based on all types of pesticides applied between harvest n-1 and harvest n. |
|  |  | **Mean tillage intensity** | Mean based on the 4-year crop sequence of the number of deep and shallow tillage interventions between harvest n-1 and harvest n. |
|  |  | **CV tillage intensity** | Coefficient of variation (CV) based on the 4-year crop sequence of the number of deep and shallow tillage interventions between harvest n-1 and harvest n. |
|  |  | **Crop sequence homogeneity** | Opposite of the diversity of the 4-year crop sequence based on the phenology of the crop (either winter, summer or temporary grassland) using the Shannon index (Shannon index multiplied by -1). |
|  | Landscape | **Mean proportion of cropland** | Mean based on the 4-year crop sequence of the proportion of the cropland area in the 1 km² surrounding landscape. |
|  |  | **Mean patch size** | Mean of the patch size area in the 1 km² surrounding landscape. Based on all types of patches. Adjoining similar crops are considered as one single patch. Categories considered for the patches are “Semi-natural habitat”, “spring cereal”, “grassland”, “perennial crops”, “legume”, “winter pulse crops”, “winter oilseed crops”, “winter cereal”, “fodder”, summer crops”, “other crops”, “others”. |
|  |  | **Mean crop phenology homogeneity** | The opposite of the mean crop Shannon index calculated at the landscape level. Values are based on three types of crops (summer, winter crops and temporary grassland). A high value reflects homogenisation of the crops sown. |
|  |  | **Variation of crop phenology homogeneity** | The opposite of the mean crop Shannon index calculated at the landscape level. Values are based on three types of crops (summer, winter crops and temporary grassland). Finally not used in the SEM because of high correlation with its mean. |
| Biotic | Carabid community | **Mean alpha richness:**  **Mean ɑ-richness** | Mean based on the 4-year crop sequence of annual α-richness. |
|  |  | **Variation of alpha richness: SD ɑ-richness** | Standard Deviation (SD) based on the 4-year crop sequence of annual α-richness. |
|  |  | **Gamma richness:**  **ɣ-richness** | Total number of species detected in a field based on the 4-year sequence. Presented in the sensitivity analyses only. |
|  |  | **Beta-diversity: ꞵ-diversity **** | Temporal beta-diversity based on the 4-year crop sequence. Calculated using the ‘beta.multi’ function (SORENSEN index) of the ‘betapart’ package (Baselga et al., 2023). |
|  |  | **Asynchrony***** | \| Ability of the community to respond to different environmental conditions. Opposite of the synchrony (Gross et al., 2014) and independent of the species richness. Values were calculated using the ‘synchrony’ function of the ‘codyn’ package (Hallett et al., 2020). To convert synchrony into asynchrony, we multiplied the synchrony by -1. \| \| --- \| |
| Functioning | Carabid biomass | **Carabid biomass mean value** | Mean of total carabid biomass based on the annual carabid biomass of the 4-year crop sequence. |
|  |  | **Carabid biomass stability (μ/σ)** | First version of the stability of total carabid biomass based on the annual carabid biomass of the 4-year crop sequence. Stability is the mean divided by the standard deviation of the mean carabid biomass. Presented in the main text |
|  |  | **Carabid biomass stability (σ/μ)^-1^** | Second version of the stability of the total carabid biomass based on the annual carabid biomass of the 6-year crop sequence. Opposite of the coefficient of variation, as described in Blüthgen et al. (2016). Presented in the sensitivity analyses only. |

*** Treatment frequency index (TFI)**

where Di is the applied dose, Si the treated surface area, Dri the reference dose obtained from the French Ministry of Agriculture database (Ephy website; https://ephy.anses.fr/) and S is the total area of the field for each spraying operation i.

$TFI=\sum_{i=1}^{n} \frac{D_{i}* S_{i}}{D_{ri}* S}$ ,

****Temporal turn-over of the community or β-diversity**

T is the number of sites in Baselga (2010). Here, T is the number of sampling years for a given field.

Bij, bji are the number of species exclusive to sites I and j, respectively when compared by pairs.

S_i_ is the total number of species in site I, S_T_ is the total number of species in all sites considered together.

$$\beta_{SOR}= \frac{[\sum_{i<j} min(b_{ij},b_{ji})]+[\sum_{i<j} max(b_{ij},b_{ji})]}{2[\sum_{i} S_{i}-S_{T}]+[\sum_{i<j} min(b_{ij},b_{ji})]+[\sum_{i<j} max(b_{ij},b_{ji})]}$$

*****Asynchrony**

According to Gross et al., 2014: “this metric is the average across species of the correlation between the biomass of each species and the total biomass of all the other species in the group. Y_i_ is the biomass of species I in a group of n species.” The advantage of this metric is that is uncorrelated to species richness. η is the synchrony metric multiplied by -1.

$$\eta=\left( \frac{1}{n} \right)\sum_{i} corr(Y_{i}, \sum_{j\neq i} Y_{j})$$

**Table S2. Hypotheses supporting the conceptual framework**

| **Hypothesis** | **Predictor** | **Hypotheses** | **Interesting references (not exhaustive)*** |
| --- | --- | --- | --- |
| H1 | Field and landscape-level management intensity | The mean intensity of the field- and landscape-level management intensities reduces the in-field mean α-richness | Chaplin-Kramer et al., 2011 ; Tuck et al., 2014 ; Henneron et al., 2015 ; Trichard et al., 2013 ; Woodcock et al., 2010 (on natural enemies or carabids) ; Alignier et al., 2020 (on plants). Some papers do not strictly support this relationship (Muneret et al., 2023) |
|  |  |  |  |
| H2 | Field and landscape-level management temporal variability* | The temporal variability of the field management intensity increases the temporal variability of the in-field α-richness, asynchrony and temporal β-diversity. | Bertrand et al., 2016 ; Bosem Baillod, 2017 ; Marta et al., 2021 ; Brown et al., 2016. Some papers show the opposite (Van Klink et al., 2019; Baselga et al., 2015) |
|  |  |  |  |
| H3 | Mean α-richness | The mean α-richness increases β-diversity, asynchrony and carabid biomass mean value and stability | Xu et al., 2021 ; Craven et al., 2018 ; Doak , 1998. The relationship between α-richness and β-diversity is not clear; Olivier et al., 2020 |
|  |  |  |  |
| H4 | SD α-richness | The temporal variability of the α-richness decreases asynchrony and the β-diversity | We found no paper about this relationship |
|  |  |  |  |
| H5 | Temporal β-diversity | The temporal β-diversity increases carabid biomass mean value and stability | We found no paper about this relationship but there some papers on the effect of spatial beta-diversityv(i.e. van de Plas et al., 2023) |
|  |  |  |  |
| H6 | Asynchrony | The asynchrony increases carabid biomass mean value and stability | Valencia et al., 2020; Xu et al., 2021 ; Olivier et al., 2020 |
|  |  |  |  |
| H7 | Carabid biomass mean value | Carabid biomass mean value increases or is positively correlated with carabid biomass stability | Craven et al., 2018; Montoya et al., 2021 (it shows a trade-off and not on carabids); Cardinale et al., 2013 |
|  |  |  |  |

*

- Alignier, A., Solé-Senan, X. O., Robleño, I., Baraibar, B., Fahrig, L., Giralt, D., Gross, N., Martin, J.-L., Recasens, J., Sirami, C., Siriwardena, G., Bosem Baillod, A., Bertrand, C., Carrié, R., Hass, A., Henckel, L., Miguet, P., Badenhausser, I., Baudry, J., … Batáry, P. (2020). Configurational crop heterogeneity increases within-field plant diversity. *Journal of Applied Ecology*, *57*(4), 654–663. https://doi.org/10.1111/1365-2664.13585
- Baselga, A., Bonthoux, S., & Balent, G. (2015). Temporal beta diversity of bird assemblages in agricultural landscapes: Land cover change vs. stochastic processes. *PLoS One*, *10*(5), e0127913.
- Bosem Baillod, A., Tscharntke, T., Clough, Y., & Batáry, P. (2017). Landscape-scale interactions of spatial and temporal cropland heterogeneity drive biological control of cereal aphids. *Journal of Applied Ecology*, *54*(6), 1804–1813.
- Brown, B. L., Downing, A. L., & Leibold, M. A. (2016). Compensatory dynamics stabilize aggregate community properties in response to multiple types of perturbations. *Ecology*, *97*(8), 2021–2033. <https://doi.org/10.1890/15-1951.1>
- Cardinale, B. J., Gross, K., Fritsche, K., Flombaum, P., Fox, J. W., Rixen, C., van Ruijven, J., Reich, P.B., Scherer-Lorenzen, M., Wilsey, B.J. (2013). Biodiversity simultaneously enhances the production and stability of community biomass, but the effects are independent. *Ecology*, 94(8), 1697-1707.
- Chaplin-Kramer, R., O’Rourke, M. E., Blitzer, E. J., & Kremen, C. (2011). A meta-analysis of crop pest and natural enemy response to landscape complexity. *Ecology Letters*, *14*(9), 922–932.
- Craven, D., Eisenhauer, N., Pearse, W. D., Hautier, Y., Isbell, F., Roscher, C., Bahn, M., Beierkuhnlein, C., Bönisch, G., & Buchmann, N. (2018). Multiple facets of biodiversity drive the diversity–stability relationship. *Nature Ecology & Evolution*, *2*(10), 1579–1587.
- Doak, D. F., Bigger, D., Harding, E. K., Marvier, M. A., O’Malley, R. E., & Thomson, D. (1998). The Statistical Inevitability of Stability‐Diversity Relationships in Community Ecology. *The American Naturalist*, *151*(3), 264–276. https://doi.org/10.1086/286117
- Henneron, L., Bernard, L., Hedde, M., Pelosi, C., Villenave, C., Chenu, C., Bertrand, M., Girardin, C., & Blanchart, E. (2015). Fourteen years of evidence for positive effects of conservation agriculture and organic farming on soil life. *Agronomy for Sustainable Development*, *35*(1), 169–181. https://doi.org/10.1007/s13593-014-0215-8
- Marta, S., Brunetti, M., Manenti, R., Provenzale, A., & Ficetola, G. F. (2021). Climate and land-use changes drive biodiversity turnover in arthropod assemblages over 150 years. *Nature Ecology & Evolution*, *5*(9), 1291–1300.
- Olivier, T., Thébault, E., Elias, M., Fontaine, B., & Fontaine, C. (2020). Urbanization and agricultural intensification destabilize animal communities differently than diversity loss. *Nature Communications*, *11*(1), 2686.
- van der Plas, F., Hennecke, J., Chase, J. M., van Ruijven, J., Barry, K. E. (2023). Universal beta-diversity-functioning relationships are neither observed nor expected. *Trends in Ecology and Evolution*, *8*(6), 532–544.
- Trichard, A., Alignier, A., Biju-Duval, L., & Petit, S. (2013). The relative effects of local management and landscape context on weed seed predation and carabid functional groups. *Basic and Applied Ecology*, *14*(3), 235–245. https://doi.org/10.1016/j.baae.2013.02.002
- Tuck, S. L., Winqvist, C., Mota, F., Ahnström, J., Turnbull, L. A., & Bengtsson, J. (2014). Land‐use intensity and the effects of organic farming on biodiversity: A hierarchical meta‐analysis. *Journal of Applied Ecology*, *51*(3), 746–755. https://doi.org/10.1111/1365-2664.12219
- Valencia, E., De Bello, F., Galland, T., Adler, P. B., Lepš, J., E-Vojtkó, A., van Klink, R., Carmona, C. P., Danihelka, J., & Dengler, J. (2020). Synchrony matters more than species richness in plant community stability at a global scale. *Proceedings of the National Academy of Sciences*, *117*(39), 24345–24351.
- van Klink, R., Lepš, J., Vermeulen, R., & de Bello, F. (2019). Functional differences stabilize beetle communities by weakening interspecific temporal synchrony. *Ecology*, *100*(8), e02748. https://doi.org/10.1002/ecy.2748
- Xu, Q., Yang, X., Yan, Y., Wang, S., Loreau, M., & Jiang, L. (2021). Consistently positive effect of species diversity on ecosystem, but not population, temporal stability. *Ecology Letters*, *24*(10), 2256–2266.

**Table S3. Number of specimens for each species sampled in each area.**

|  | Dijon | Rennes | Toulouse | Diet |
| --- | --- | --- | --- | --- |
| Abax parallelepipedus | 1 | 0 | 0 | Carnivorous |
| Abax parallelus | 3 | 0 | 0 | Carnivorous |
| Acupalpus dubius | 1 | 1 | 0 | NA |
| Acupalpus elegans | 0 | 2 | 0 | NA |
| Acupalpus meridianus | 4 | 6 | 6 | Omnivorous |
| Agonum afrum | 0 | 1 | 0 | NA |
| Agonum muelleri | 48 | 15 | 11 | Carnivorous |
| Agonum sexpunctatum | 1 | 0 | 0 | Carnivorous |
| Agonum sp. | 1 | 0 | 0 | NA |
| Agonum viduum | 0 | 1 | 0 | NA |
| Amara aenea | 44 | 24 | 4 | Phytophagous |
| Amara communis | 0 | 3 | 0 | Phytophagous |
| Amara consularis | 3 | 0 | 0 | Phytophagous |
| Amara convexior | 1 | 0 | 0 | Phytophagous |
| Amara familiaris | 1 | 3 | 0 | Phytophagous |
| Amara fulvipes | 2 | 0 | 0 | Phytophagous |
| Amara lunicollis | 0 | 1 | 0 | NA |
| Amara ovata | 36 | 1 | 1 | Phytophagous |
| Amara plebeja | 3 | 13 | 0 | NA |
| Amara similata | 307 | 162 | 7 | Phytophagous |
| Anchomenus dorsalis | 571 | 391 | 697 | Carnivorous |
| Anisodactylus binotatus | 15 | 7 | 1 | Phytophagous |
| Anisodactylus signatus | 277 | 0 | 0 | Omnivorous |
| Asaphidion gr. flavipes | 4 | 14 | 0 | Carnivorous |
| Badister bullatus | 1 | 1 | 1 | Carnivorous |
| Badister sodalis | 5 | 0 | 0 | NA |
| Bembidion quadrimaculatum | 18 | 82 | 1 | Carnivorous |
| Brachinus crepitans | 2128 | 61 | 346 | Omnivorous |
| Brachinus elegans | 51 | 0 | 14 | NA |
| Brachinus explodens | 7 | 53 | 42 | Carnivorous |
| Brachinus immaculicornis | 0 | 0 | 3 | NA |
| Brachinus sclopeta | 879 | 447 | 9 | Omnivorous |
| Bradycellus distinctus | 0 | 0 | 1 | Omnivorous |
| Bradycellus harpalinus | 0 | 2 | 0 | Omnivorous |
| Calathus fuscipes | 2 | 1 | 7 | Carnivorous |
| Carabus auratus | 26 | 37 | 233 | Carnivorous |
| Carabus cancellatus | 0 | 0 | 47 | Carnivorous |
| Carabus convexus | 12 | 0 | 0 | Carnivorous |
| Carabus monilis | 1 | 0 | 0 | Carnivorous |
| Carabus nemoralis | 0 | 1 | 1 | Carnivorous |
| Carabus violaceus | 0 | 0 | 8 | Carnivorous |
| Chlaeniellus nigricornis | 3 | 0 | 1 | Carnivorous |
| Chlaeniellus olivieri | 0 | 0 | 3 | NA |
| Chlaenius chrysocephalus | 0 | 0 | 38 | NA |
| Cicindela campestris | 0 | 0 | 2 | Carnivorous |
| Clivina gr. fossor | 7 | 0 | 0 | Carnivorous |
| Cylindera germanica | 3 | 0 | 0 | Carnivorous |
| Demetrias atricapillus | 2 | 11 | 7 | Carnivorous |
| Diachromus germanus | 118 | 1 | 6 | Phytophagous |
| Drypta dentata | 1 | 0 | 0 | Carnivorous |
| Gynandromorphus etruscus | 0 | 0 | 3 | NA |
| Harpalus affinis | 170 | 57 | 5 | Phytophagous |
| Harpalus atratus | 1 | 0 | 0 | Phytophagous |
| Harpalus cupreus | 0 | 0 | 7 | Phytophagous |
| Harpalus dimidiatus | 132 | 0 | 63 | Phytophagous |
| Harpalus distinguendus | 65 | 4 | 38 | Phytophagous |
| Harpalus flavescens | 0 | 1 | 0 | Phytophagous |
| Harpalus latus | 1 | 0 | 0 | Phytophagous |
| Harpalus oblitus | 0 | 0 | 18 | Phytophagous |
| Harpalus rubripes | 4 | 3 | 0 | Phytophagous |
| Harpalus rufipes | 278 | 13 | 72 | Phytophagous |
| Harpalus smaragdinus | 1 | 0 | 0 | Phytophagous |
| Harpalus sp. | 0 | 0 | 1 | Phytophagous |
| Harpalus tardus | 1 | 0 | 0 | Phytophagous |
| Laemostenus terricola | 1 | 0 | 0 | NA |
| Leistus ferrugineus | 1 | 0 | 0 | Carnivorous |
| Leistus montanus | 0 | 0 | 1 | Carnivorous |
| Limodromus assimilis | 1 | 0 | 0 | Carnivorous |
| Loricera pilicornis | 37 | 19 | 0 | Carnivorous |
| Metallina lampros | 60 | 233 | 15 | Carnivorous |
| Metallina properans | 38 | 8 | 9 | Carnivorous |
| Microlestes maurus | 1 | 3 | 0 | Carnivorous |
| Microlestes minutulus | 3 | 3 | 0 | Carnivorous |
| Microlestes sp. | 9 | 0 | 9 | NA |
| Nebria brevicollis | 29 | 57 | 15 | Carnivorous |
| Nebria salina | 165 | 32 | 19 | Carnivorous |
| Notiophilus aquaticus | 2 | 0 | 0 | Carnivorous |
| Notiophilus biguttatus | 3 | 0 | 0 | Carnivorous |
| Notiophilus palustris | 0 | 2 | 0 | Carnivorous |
| Notiophilus quadripunctatus | 5 | 13 | 0 | Carnivorous |
| Ocys quinquestriatus | 0 | 0 | 5 | NA |
| Ophonus ardosiacus | 0 | 0 | 6 | Carnivorous |
| Ophonus azureus | 1 | 0 | 2 | Phytophagous |
| Ophonus laticollis | 0 | 1 | 0 | NA |
| Ophonus puncticeps | 0 | 2 | 0 | Phytophagous |
| Ophonus sabulicola | 0 | 0 | 2 | Phytophagous |
| Ophonus subg. Metophonus | 1 | 1 | 0 | NA |
| Paratachys bistriatus | 1 | 0 | 0 | NA |
| Parophonus maculicornis | 0 | 1 | 0 | NA |
| Parophonus mendax | 1 | 0 | 9 | Phytophagous |
| Philochthus biguttatus | 0 | 11 | 0 | Carnivorous |
| Philochthus iricolor | 1 | 0 | 6 | Carnivorous |
| Philochthus lunulatus | 0 | 0 | 4 | Carnivorous |
| Phyla obtusa | 17 | 262 | 17 | Carnivirous |
| Poecilus cupreus | 2464 | 976 | 635 | Omnivirous |
| Polistichus connexus | 1 | 0 | 0 | NA |
| Pterostichus anthracinus | 2 | 0 | 0 | Carnivorous |
| Pterostichus macer | 0 | 0 | 2 | Carnivorous |
| Pterostichus madidus | 0 | 0 | 211 | Omnivorous |
| Pterostichus melanarius | 721 | 771 | 0 | Carnivorous |
| Pterostichus strenuus | 0 | 1 | 0 | Carnivorous |
| Pterostichus vernalis | 4 | 1 | 0 | Carnivorous |
| Scybalicus oblongiusculus | 23 | 0 | 4 | NA |
| Semiophonus signaticornis | 8 | 1 | 0 | Phytophagous |
| Sinechostictus stomoides | 0 | 0 | 1 | Carnivorous |
| Stenolophus skrimshiranus | 0 | 0 | 2 | Omnivorous |
| Stenolophus teutonus | 7 | 0 | 1 | Omnivorous |
| Stomis pumicatus | 0 | 1 | 1 | Carnivorous |
| Syntomus obscuroguttatus | 3 | 19 | 0 | Carnivorous |
| Syntomus truncatellus | 0 | 2 | 0 | Carnivorous |
| Trechus gr. quadristriatus | 107 | 423 | 62 | Carnivorous |
| Zabrus tenebrioides | 1 | 0 | 5 | Phytophagous |

**Table S4. Results of the Moran’s tests showing there is no spatial autocorrelation in the final models included in the piecewise structural equation models.**

| Piecewise structural Equation Model | Model | Moran I statistic standard deviate | p-value | | Moran I statistic | Expectation | Variance |
| --- | --- | --- | --- | --- | --- | --- | --- |
| PW1 | M1 : Mean α-Richness | 0,81 | | 0,21 | 0,04 | -0,02 | 0,0051 |
| Main text | M2 : β-diversity | 0,16 | | 0,44 | -0,01 | -0,02 | 0,0056 |
|  | M3 : Asynchrony | 0,21 | | 0,42 | -0,00 | -0,02 | 0,0055 |
|  | M4 : Carabid Biomass Mean Value (μ) | 0,22 | | 0,41 | -0,00 | -0,02 | 0,0052 |
|  | M5 : Carabid Biomass Stability (μ/σ) | -0,55 | | 0,72 | -0,06 | -0,02 | 0,0055 |
| PW2 | M1 : γ-Richness | -0,74 | | 0,77 | -0,07 | -0,02 | 0,0056 |
|  | M2 : β-diversity | 1,72 | | 0,04 | 0,11 | -0,02 | 0,0056 |
|  | M3 : Asynchrony | 0,43 | | 0,34 | 0,01 | -0,02 | 0,0054 |
|  | M4 : Carabid Biomass Mean Value (μ) | 0,22 | | 0,41 | -0,00 | -0,02 | 0,0052 |
|  | M5 : Carabid Biomass Stability (μ/σ) | -0,55 | | 0,72 | -0,06 | -0,02 | 0,0055 |
| PW3 | M1 : Mean α-Richness | 0,81 | | 0,21 | 0,04 | -0,02 | 0,0055 |
|  | M2 : β-diversity | 0,16 | | 0,44 | -0,01 | -0,02 | 0,0055 |
|  | M3 : Asynchrony | 0,26 | | 0,40 | 0,00 | -0,02 | 0,0054 |
|  | M4 : Carabid Biomass Mean Value (μ) | 0,22 | | 0,41 | -0,00 | -0,02 | 0,0052 |
|  | M5 : Carabid Biomass Stability (CV-1) | 1,24 | | 0,11 | 0,08 | -0,02 | 0,0057 |
| PW4 | M1 : γ-Richness | -0,74 | | 0,77 | -0,07 | -0,02 | 0,0056 |
|  | M2 : β-diversity | 1,72 | | 0,04 | 0,11 | -0,02 | 0,0055 |
|  | M3 : Asynchrony | 0,43 | | 0,34 | 0,01 | -0,02 | 0,0054 |
|  | M4 : Carabid Biomass Mean Value (μ) | 0,22 | | 0,41 | -0,00 | -0,02 | 0,0052 |
|  | M5 : Carabid Biomass Stability (CV-1) | 1,24 | | 0,11 | 0,08 | -0,02 | 0,0057 |

**
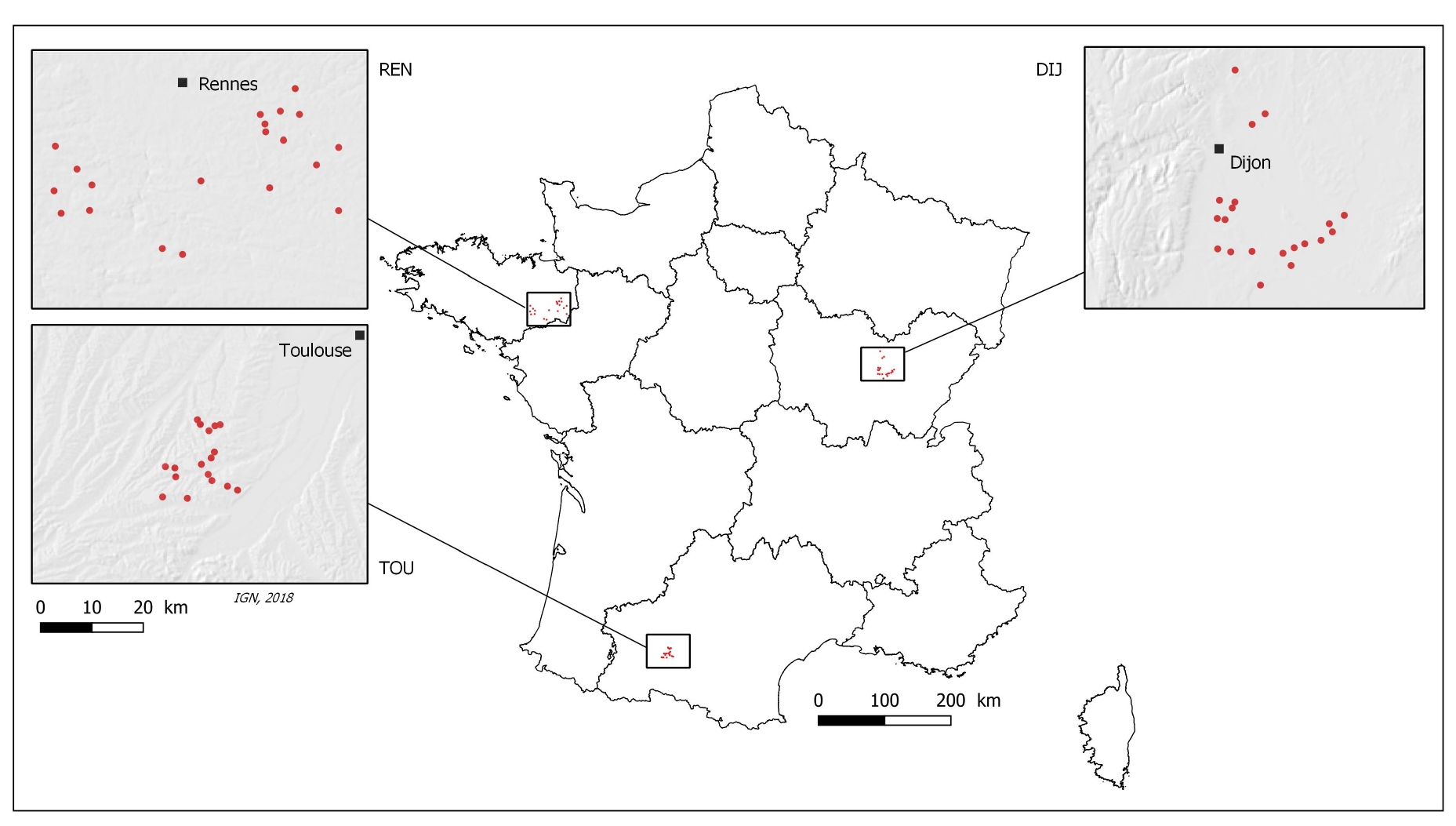
**

**Figure S1. Map of the 57 arable agroecosystems of the study.**

**
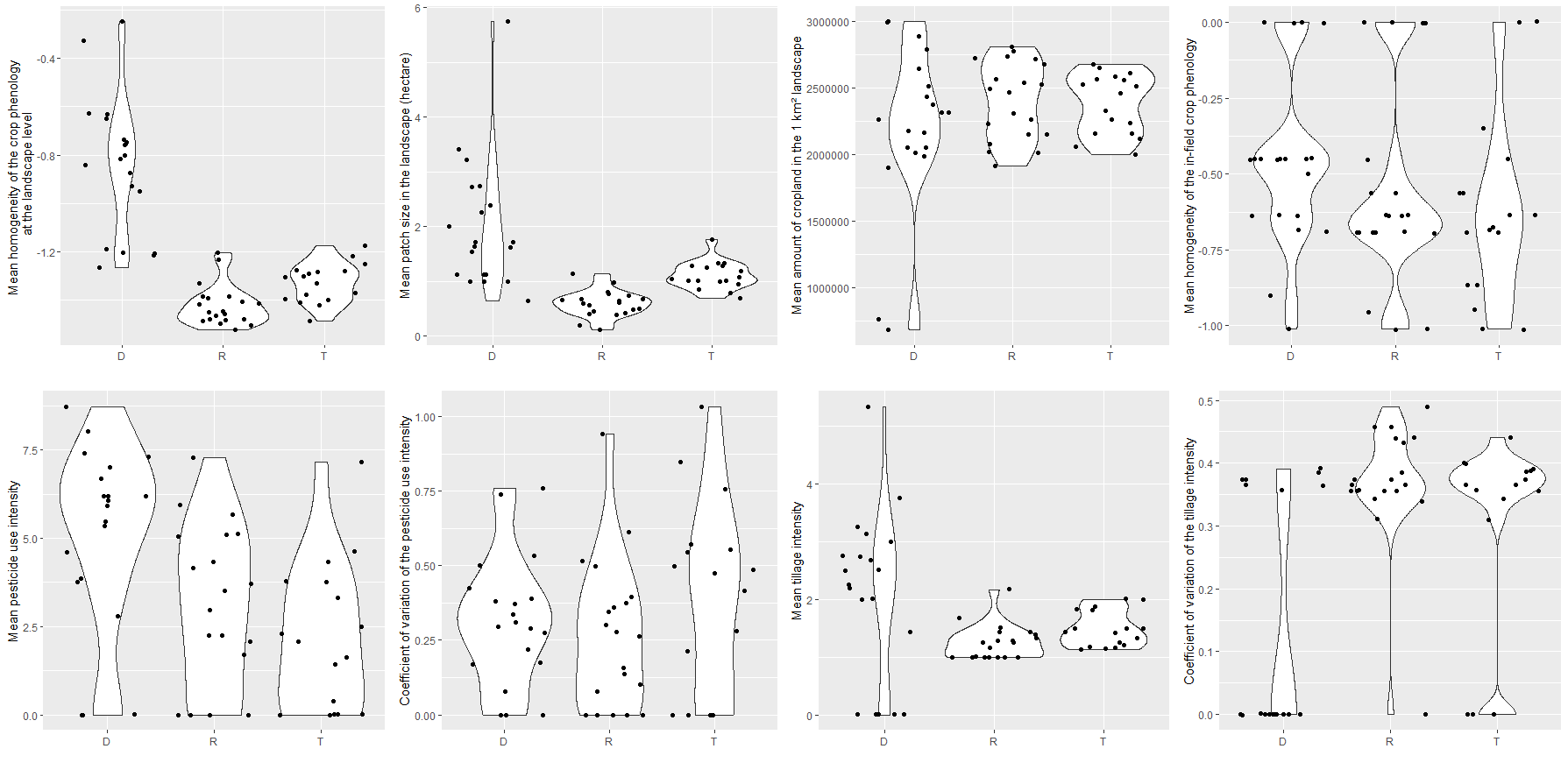
**

**Figure S2. Correlations between abiotic variables (“D”, “R” and “T” mean Dijon, Rennes and Toulouse area respectively).**


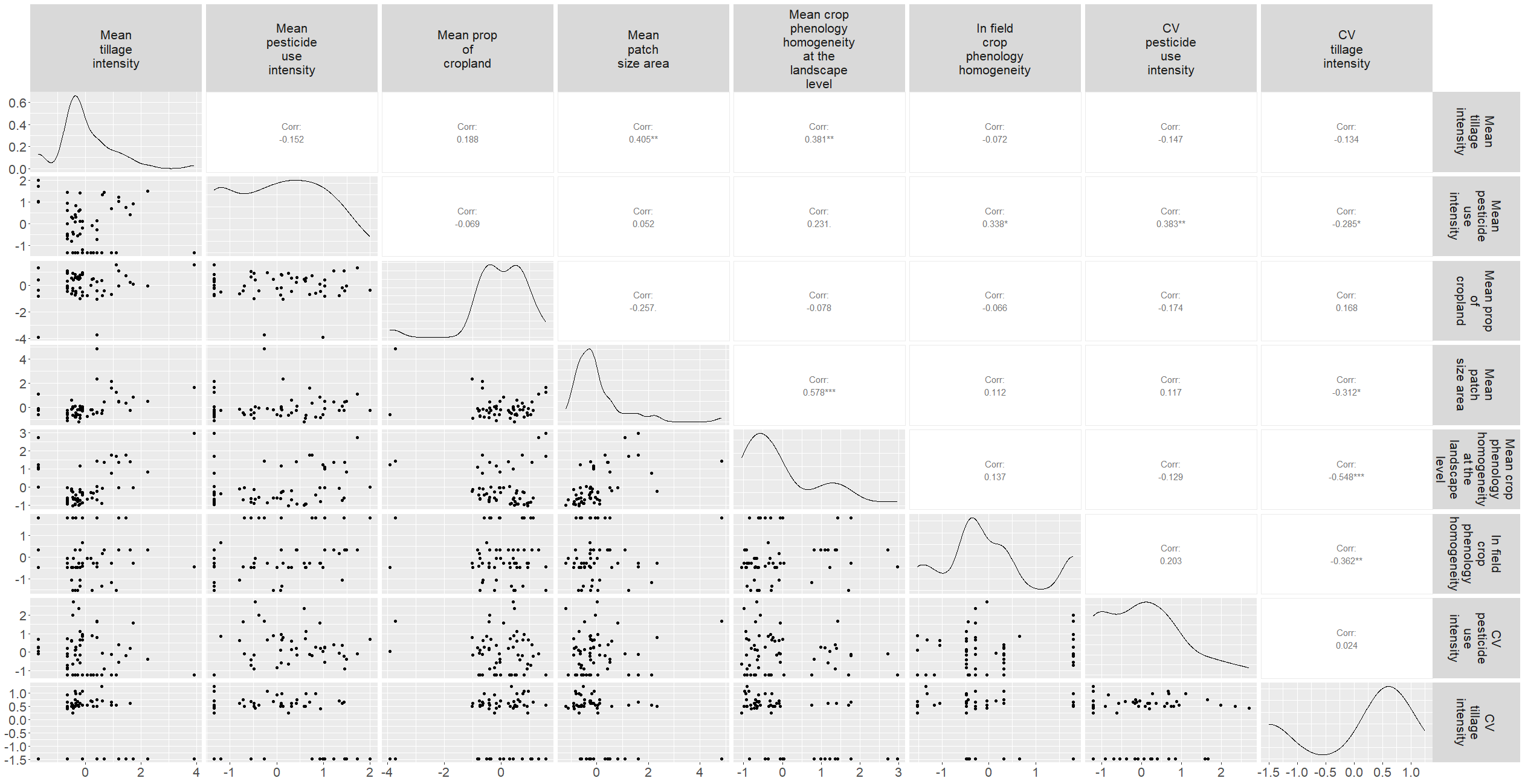


**Figure S3. Pearson correlations between the scaled abiotic variables.**

**
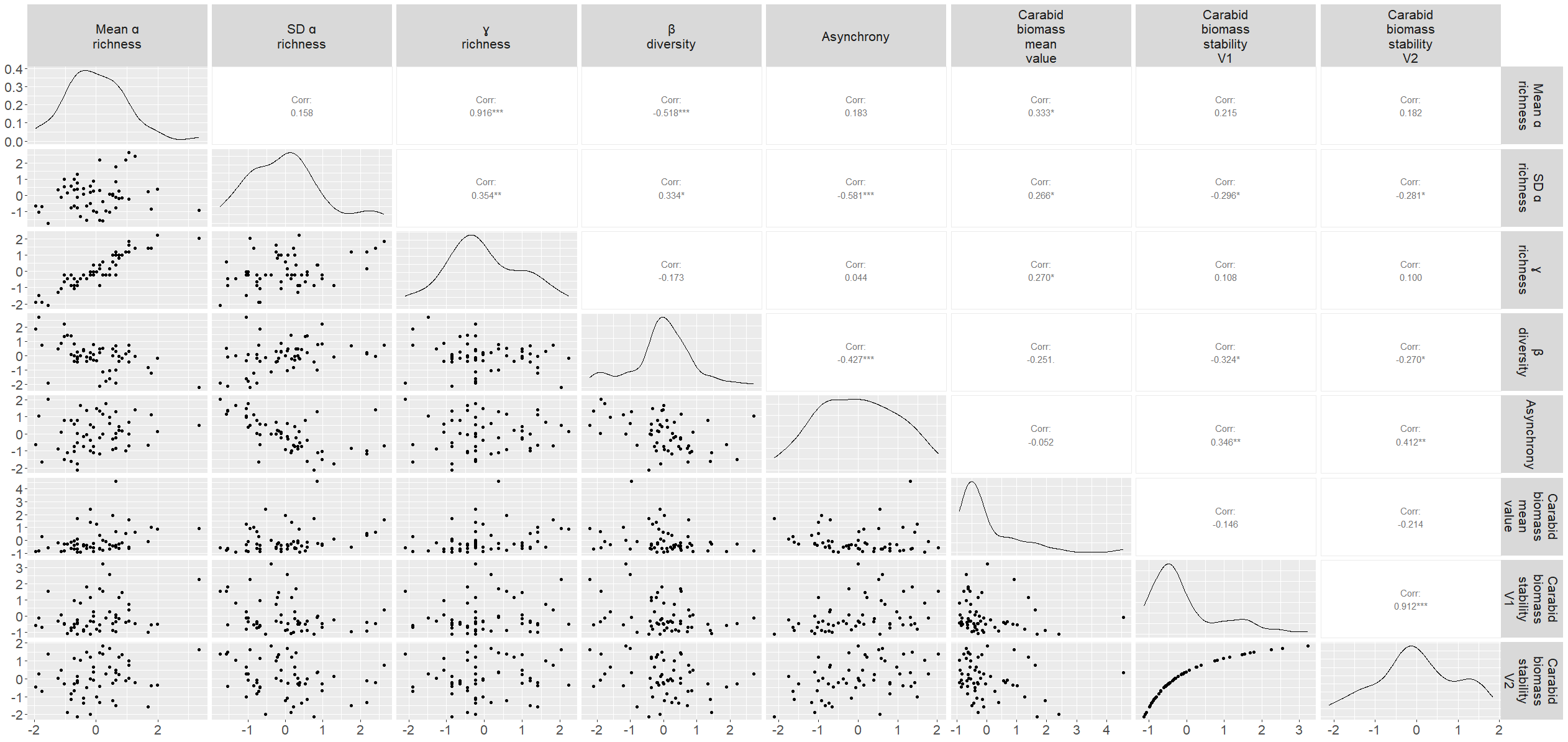
**

**Figure S4. Pearson correlations between the scaled biotic variables (richness, composition, functioning descriptors).** Carabid biomass stability V1 was calculated using (mean/SD) and V2 (SD/mean)^-1^.

**
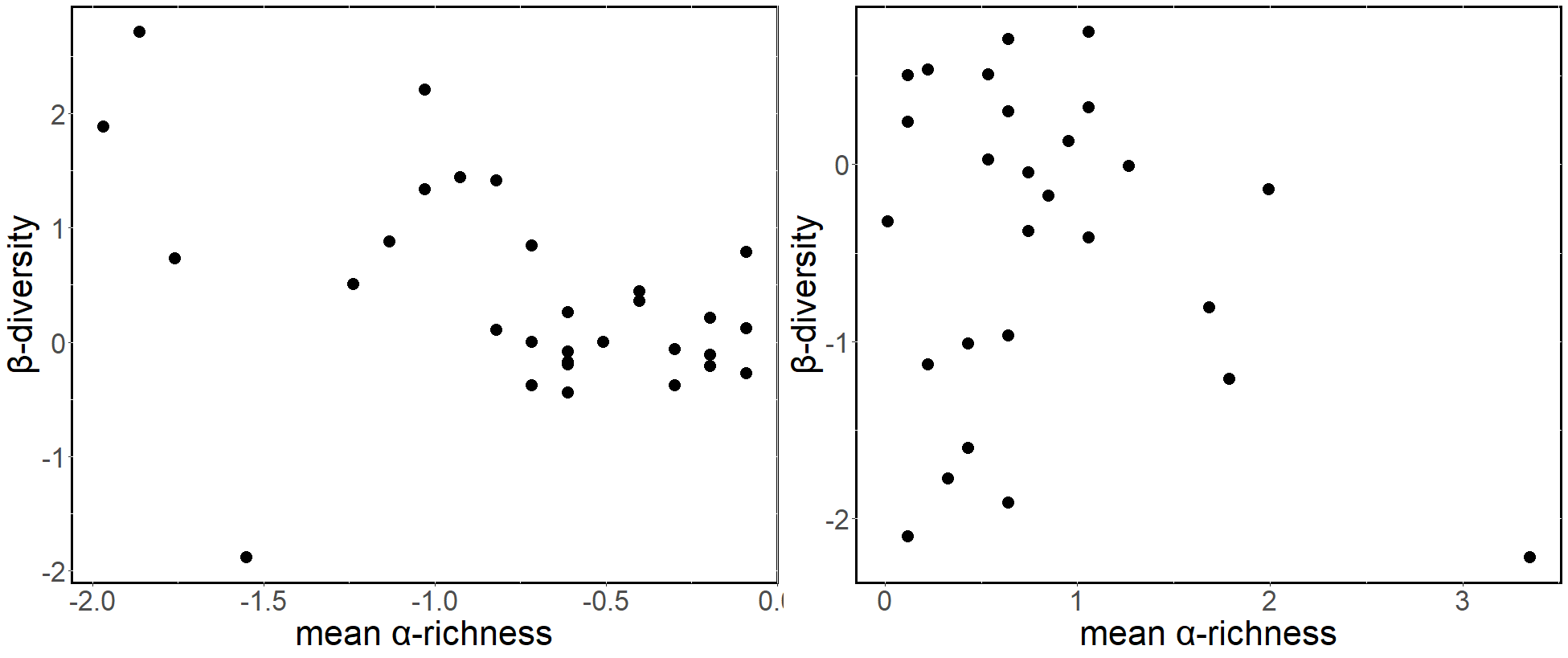
**

**Figure S5. Relationships between mean α-richness and β-diversity for the poorer and richer fields (i.e. negative and positive values for mean α-richness respectively after scaling).**

**
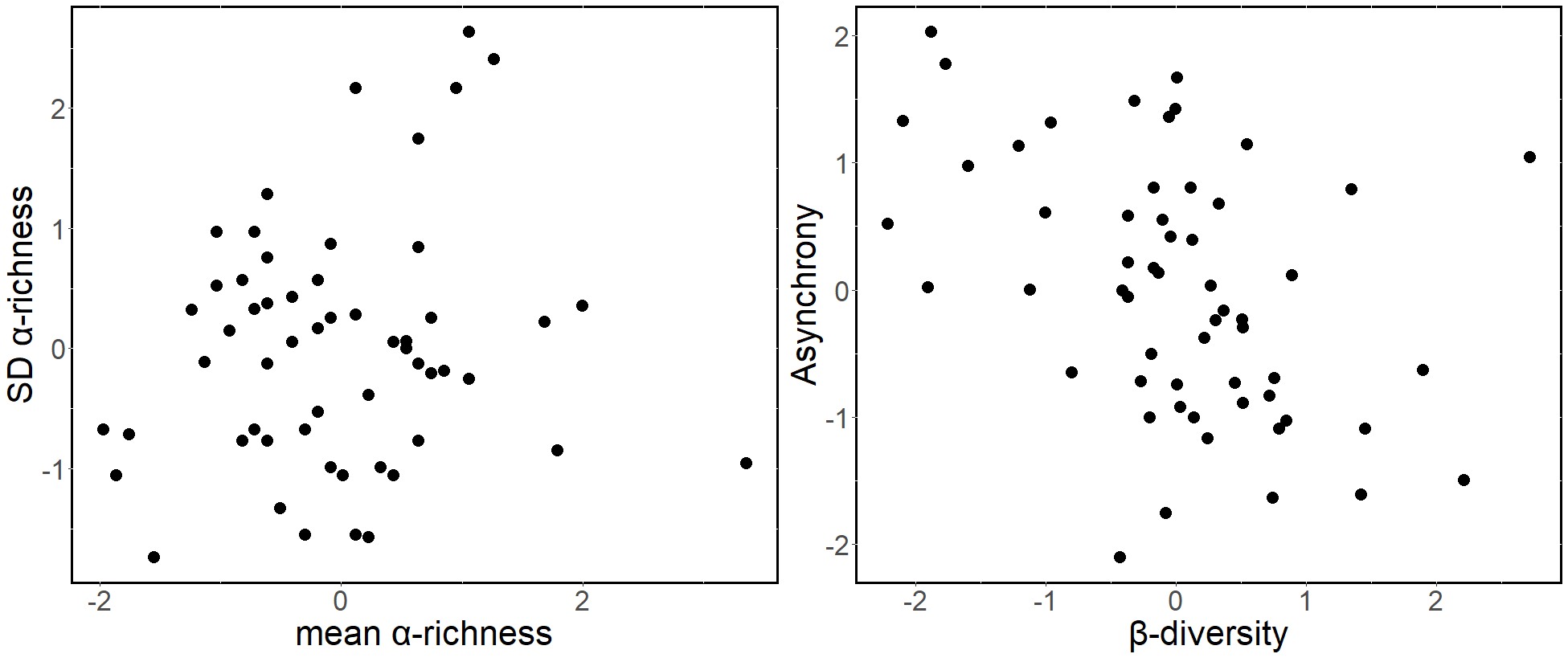
**

**Figure S6. Co-variations between (i) mean and SD α-richness and (ii) β-diversity and Asynchrony.**

**Appendix S1**

**Sampling dates for the carabid community and references used for determining and assessing the body length of the carabid species**

|  |  | **Dijon** | | **Rennes** | | **Toulouse** | |
| --- | --- | --- | --- | --- | --- | --- | --- |
|  |  | **Winter** | **Summer** | **Winter** | **Summer** | **Winter** | **Summer** |
| 2014 | Sampling date 1 | May-15 | no data | May-05 | no data | April-14 | no data |
|  | Sampling date 2 | June-12 | no data | June-02 | no data | May-19 | no data |
| 2015 | Sampling date 1 | May-28 | July-02 | May-18 | June-15 | April-29 | June-03 |
|  | Sampling date 2 | May-22 | July-16 | June-15 | no data | May-07 | July-01 |
| 2016 | Sampling date 1 | May-19 | June-30 | May-11 | June-09 | April-25 or -27 | June-06 |
|  | Sampling date 2 | June-09 | July-21 | June-09 | no data | May-23 to 26 | July-04 |
| 2017 | Sampling date 1 | May-18 | June-08 | no data | no data | April-24 | June-12 |
|  | Sampling date 2 | June-08 | June-29 | no data | no data | May-29 | July-03 |
| 2018 | Sampling date 1 | May-25 | June-14 or -21 | May-17 | June-14 | April-23 or -24 | June-04 |
|  | Sampling date 2 | June-14 or -21 | July-04 | June-14 | no data | June-01 or July-02 | July-02 |
| 2019 | Sampling date 1 | May-27 | June-24 | no data | no data | April-19 | June-07 |
|  | Sampling date 2 | June-24 | July-22 | no data | no data | May-24 | July-05 |

As this method leads to different sampling dates between areas, we checked that it does not drive to species composition (see below).

**Mean of the relative abundance of five dominant species over the sampling dates each year**


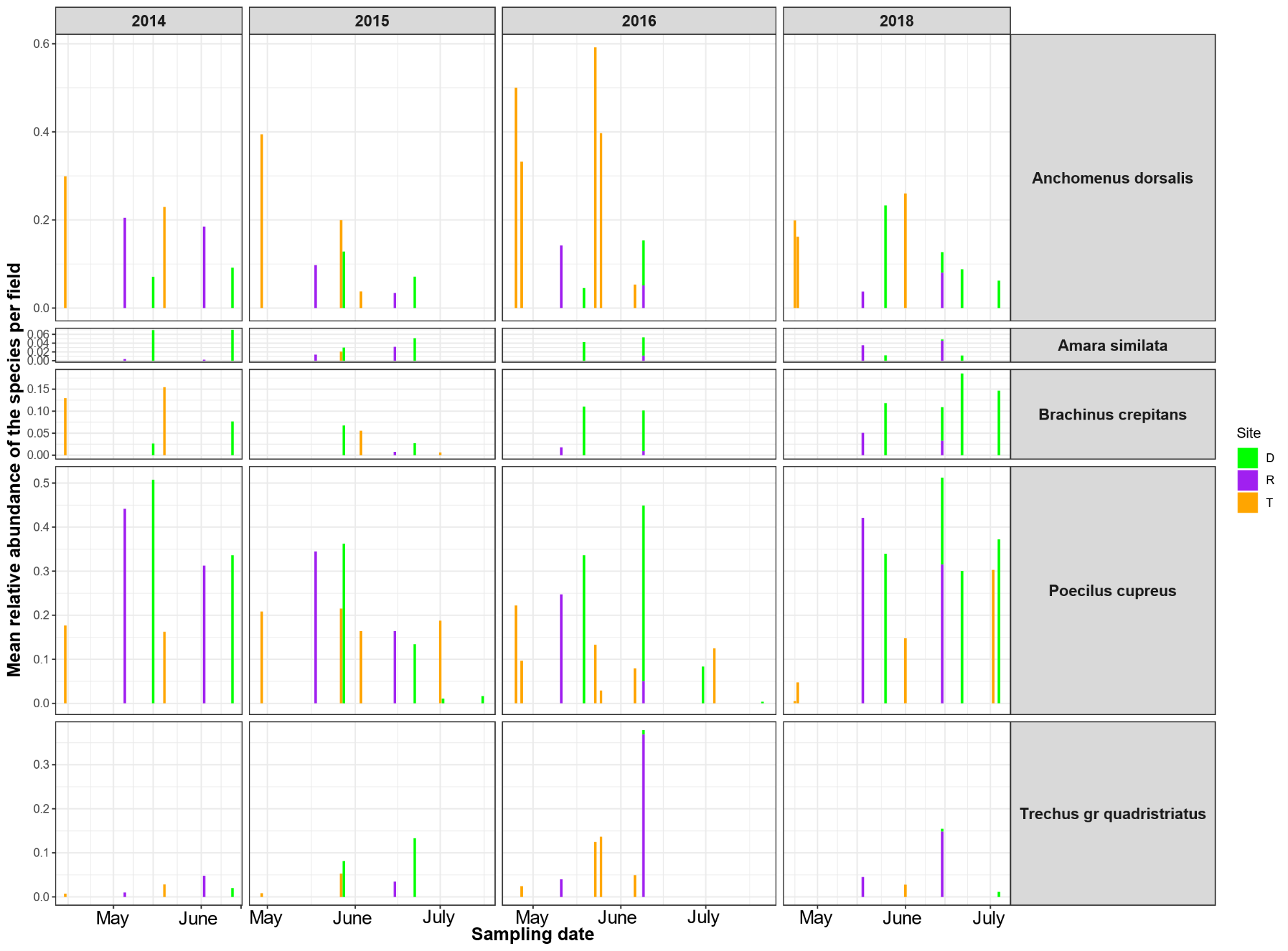


**References used to determine carabid identity.** *References used for assessing the body length of the carabid community: the mean value between the minimum and maximum values.

Roger, J., L., Jambon, O., & Boucher, J. (2016). Clé de détermination des carabidés des paysages agricoles du Nord-Ouest de la France, 256 pages. <https://www6.rennes.inrae.fr/bagap/content/download/3727/37427/version/1/file/cl%C3%A9_carabidae_nord_ouest_v6.pdf>

*Jeannel., R., (1941). Coléoptères carabiques. Faune de France 39, Première partie. 571 pages. <http://www.faunedefrance.org/bibliotheque/docs/R.%20JEANNEL(FdeFr39)%20Carab%20vol%201.pdf>

*Jeannel, R., (1942). Coléoptères carabiques. Faune de France 40, Deuxième partie. 601 pages. <http://www.faunedefrance.org/bibliotheque/docs/R.%20JEANNEL(FdeFr40)%20Carab%20vol%202%20.pdf>

* Coulon, J., Pupier, R., Quéinnec, E., Ollivier, E. & Richoux, P. (2011) Coléoptères carabiques Compléments et mise à jour. Faune de France 94, Volume 1, 352 pages. <https://faunedefrance.org/faune-94/>

*Coulon, J., Pupier, R., Quéinnec, E., Ollivier, E. & Richoux, P. (2011) Coléoptères carabiques Compléments et mise à jour. Faune de France 95, Volume 2, 337 pages. <https://faunedefrance.org/faune-95/>

**Appendix S2**

**Set of models included the initial SEM presented in the main text**. For the sensitivity analyses, the three other versions of the SEM are: 1) Mean ɑ-richness was replaced by ɣ-richness, 2) Carabid biomass stability (μ/σ) was replaced by Carabid biomass stability (σ/μ)^-1^ and 3) Mean ɑ-richness and Carabid biomass stability (μ/σ) were replaced by ɣ-richness and Carabid biomass stability (σ/μ)^-1^ respectively.

- Mean ɑ-richness ~ Mean pesticide use intensity + Mean tillage intensity + Mean proportion of cropland + Mean patch size area + Mean crop phenology homogeneity at the landscape level
- SD ɑ-richness ~ CV pesticide use intensity + CV tillage intensity + In-field crop phenology homogeneity
- ꞵ-diversity ~ CV pesticide use intensity + CV tillage intensity + + In-field crop phenology homogeneity + Mean ɑ-richness + SD ɑ-richness
- Asynchrony ~ CV pesticide use intensity + CV tillage intensity + In-field crop phenology homogeneity + Mean ɑ-richness + SD ɑ-richness
- Carabid biomass mean value ~ Mean ɑ-richness + Asynchrony + ꞵ-diversity
- Carabid biomass stability (μ/σ) ~ Mean ɑ-richness + Asynchrony + ꞵ-diversity + Carabid biomass mean value

Offset included in each model composing the SEM: Log (Number of traps used to collect carabids) and a potential co-variation link was included between Mean ɑ-richness and SD ɑ-richness.

We did not include random terms in the models to consider the three regions because this is the number of regions that lead to covering this huge diversity if landscape contexts (Figure Appendix S2).


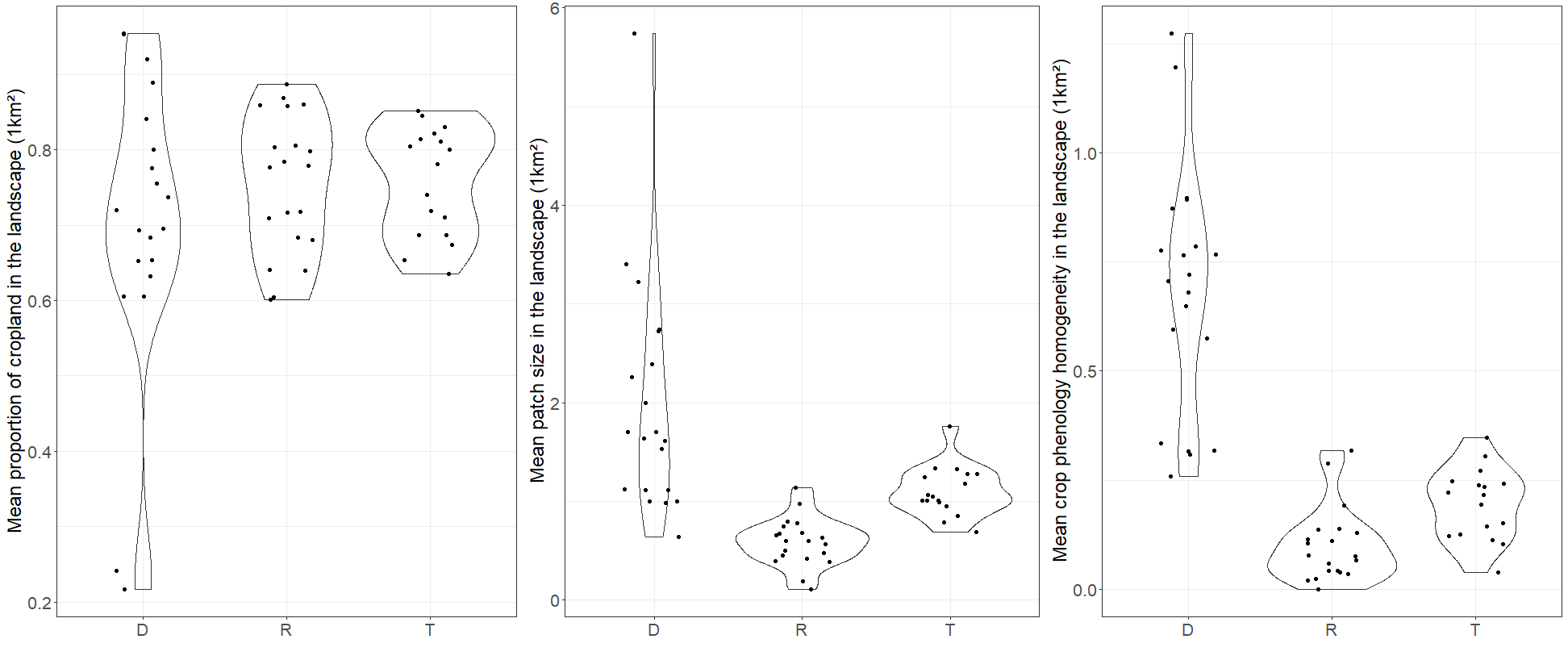


**Figure Appendix S2. Distribution of the three landscape descriptor values according to regions. D, R and T represent Dijon, Rennes and Toulouse regions respectively.**

**Appendix S3**

**Relationships between mean α-richness and A) mean proportion of cropland, B) Mean tillage intensity.** These relationships were kept in the model included mean α-richness in the final version of the PSEM presented in the main text. Both of them were significant when summing direct and indirect effects (i.e. total effects). All the variables are scaled.

**
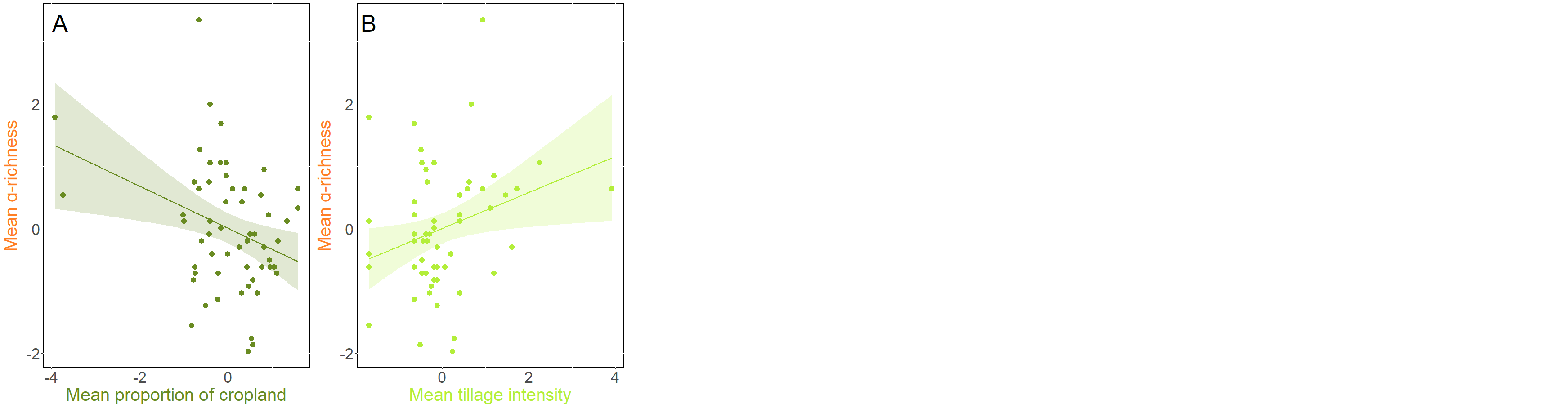
**

**Relationships between β-diversity and A) mean patch area, B) mean crop phenology homogeneity, C) mean α-richness and D) SD α-richness.** These relationships were kept in the model included mean β-diversity as response in the final version of the PSEM presented in the main text. All of them were significant when summing direct and indirect effects (i.e. total effects). All the variables are scaled.

**
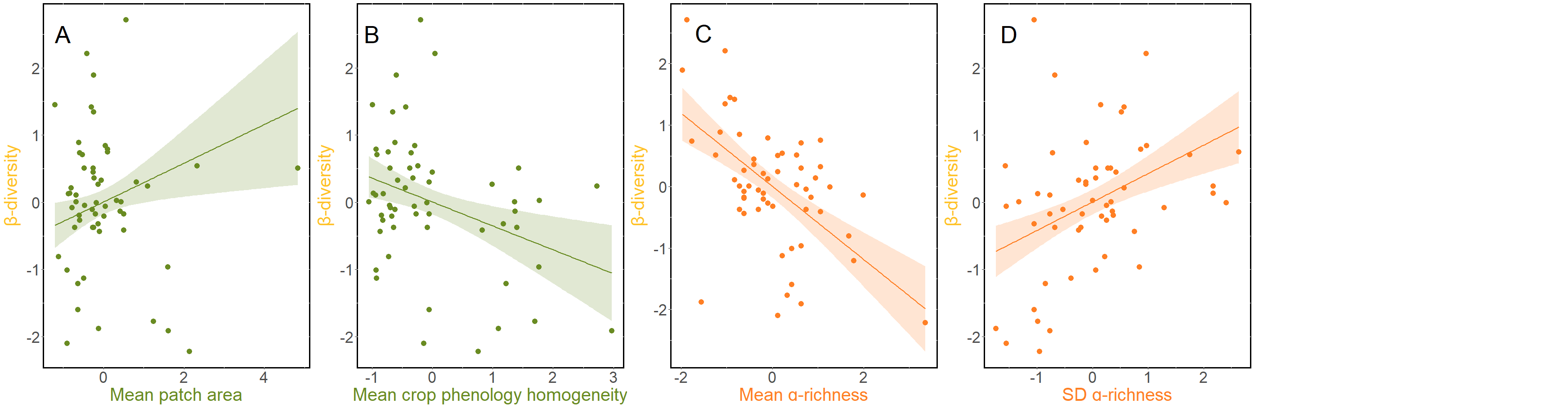
**

**Relationships between Asynchrony and A) mean crop phenology homogeneity, B) mean tillage intensity, C) CV tillage intensity, D) mean α-richness, E) SD α-richness.** These relationships were kept in the model included asynchrony as response in the final version of the PSEM presented in the main text. All of them were significant when summing direct and indirect effects (i.e. total effects) except mean crop phenology homogeneity. All the variables are scaled.

**
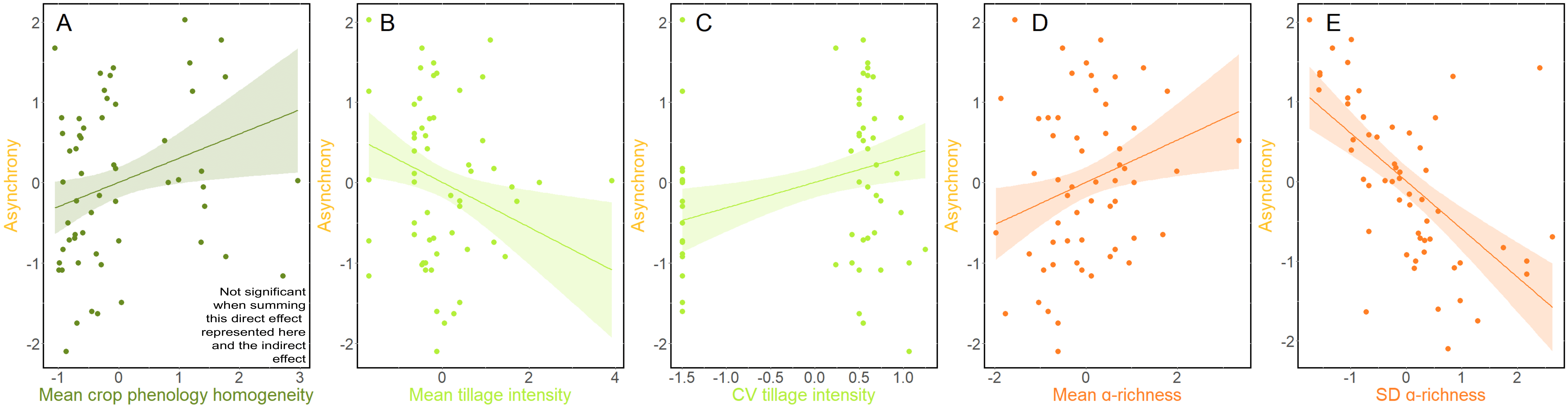
**

**Relationships between carabid biomass mean value and A) mean crop phenology homogeneity, B) CV tillage intensity, C) SD α-richness, D) β-diversity.** These relationships were kept in the model included carabid biomass mean value in the final version of the PSEM presented in the main text. All of them were significant when summing direct and indirect effects (i.e. total effects) except CV tillage intensity. All the variables are scaled.


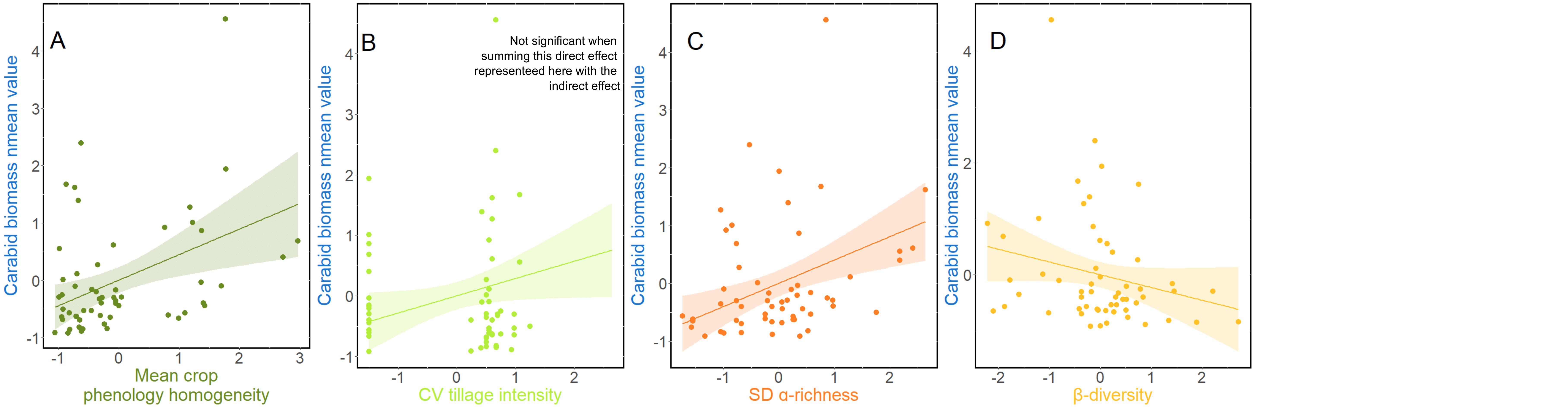


**Relationships between carabid biomass stability and A) mean crop phenology homogeneity, B) mean patch area, C) β-diversity and D) Asynchrony.** These relationships were kept in the model included carabid biomass stability in the final version of the PSEM presented in the main text. All of them were significant when summing direct and indirect effects (i.e. total effects) except asynchrony (but this latter was significant in other versions of the PSEM). All the variables are scaled.

.**
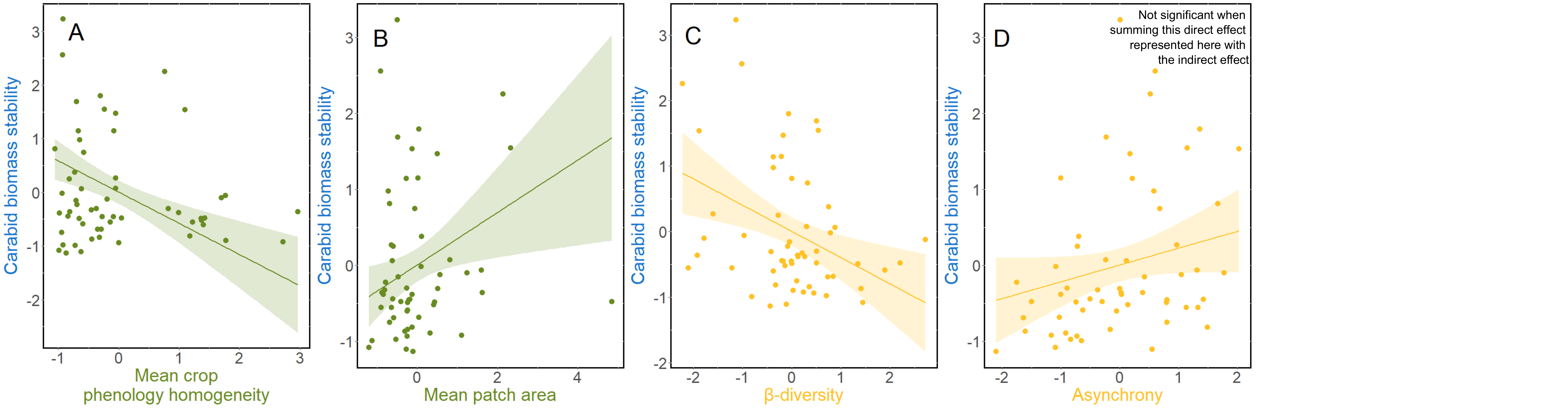
**

**Appendix S4**

**The three alternative versions of the SEM. In each of the three sections, the figures and tables are presented. A)** Using ɣ-richness instead of mean α-richness. B) Using CV-1 (σ/ μ ^-1^) as the stability metric of carabid biomass instead of μ/σ. C) Using ɣ-richness and CV-1(carabid biomass). All variables were standardised.

**SECTION A**

**Figure A Appendix S4**


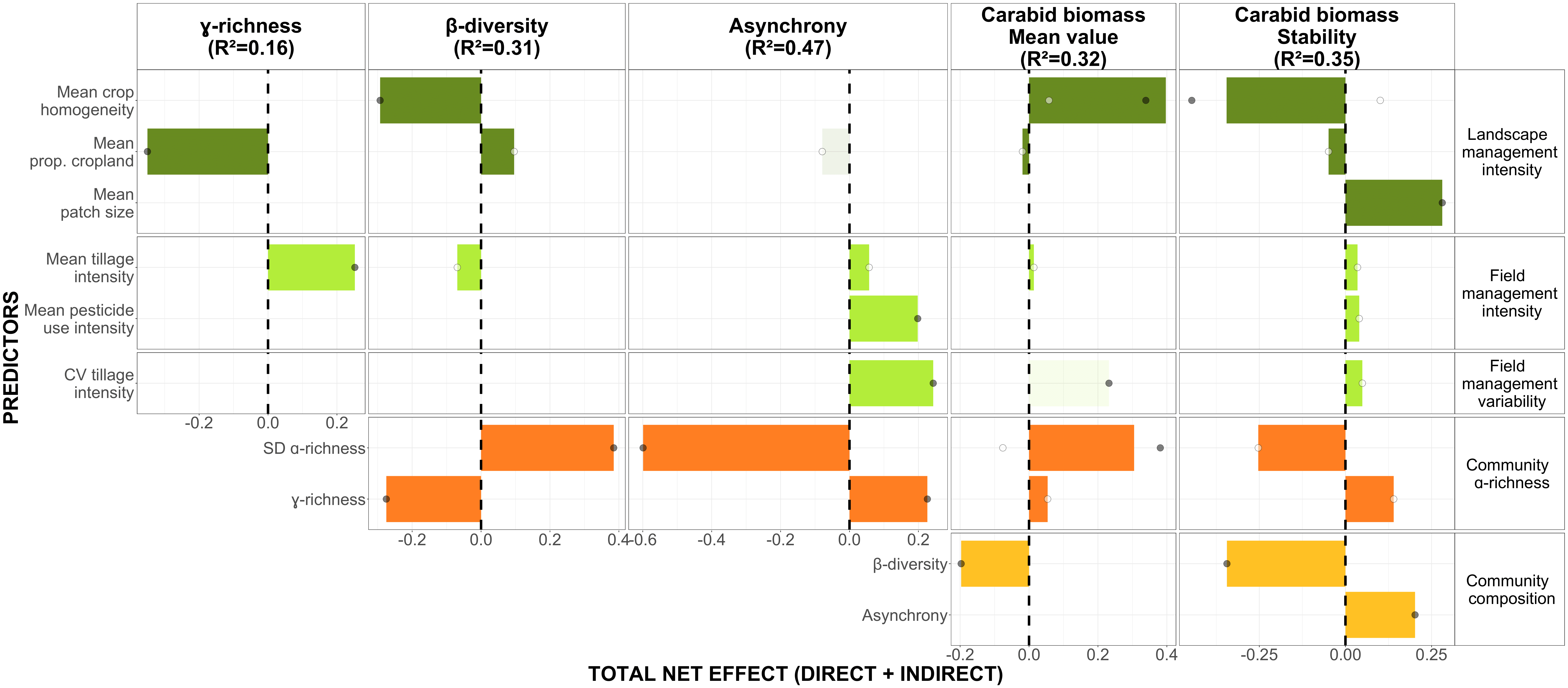


**Table A Appendix S4**

**Direct and indirect effects of the predictors based on the final version of the SEM.** Values were calculated using the semEff package (Murphy, 2022). Five models compose the SEM. Est, estimate; SE, standard error; LCI, lower confidence interval; UCI, upper confidence interval; MED, mediators. **This version includes** **ɣ-richness instead of mean α-richness in the version presented in the main text.**

| Response | Path | Predictor | Estimates | Bias | SE | LCI | UCI |
| --- | --- | --- | --- | --- | --- | --- | --- |
| γ-richness | DIRECT | **Mean prop. cropland** | **-0.350** | **-0.001** | **0.107** | **-0.494** | **-0.001** |
|  |  | **Mean tillage int.** | **0.252** | **-0.005** | **0.113** | **0.031** | **0.431** |
|  | TOTAL | **Mean prop. cropland** | **-0.350** | **-0.001** | **0.107** | **-0.494** | **-0.001** |
|  |  | **Mean tillage intensity** | **0.252** | **-0.005** | **0.113** | **0.031** | **0.431** |
| β-diversity | DIRECT | **Mean crop pheno. homog.** | **-0.293** | **0.016** | **0.101** | **-0.518** | **-0.140** |
|  |  | **SD α richness** | **0.385** | **-0.009** | **0.102** | **0.169** | **0.552** |
|  |  | **γ richness** | **-0.275** | **0.001** | **0.102** | **-0.472** | **-0.029** |
|  | INDIRECT | **Mean prop. cropland** | **0.096** | **0.001** | **0.048** | **0.006** | **0.194** |
|  |  | **Mean tillage int.** | **-0.069** | **0.005** | **0.037** | **-0.159** | **-0.021** |
|  | TOTAL | **Mean prop. cropland** | **0.096** | **0.001** | **0.048** | **0.006** | **0.194** |
|  |  | **Mean tillage int.** | **-0.069** | **0.005** | **0.037** | **-0.159** | **-0.021** |
|  |  | **Mean crop pheno. homog.** | **-0.293** | **0.016** | **0.101** | **-0.518** | **-0.140** |
|  |  | **SD α richness** | **0.385** | **-0.009** | **0.102** | **0.169** | **0.552** |
|  |  | **γ richness** | **-0.275** | **0.001** | **0.102** | **-0.472** | **-0.029** |
|  | MED. | γ richness | 0.027 | 0.006 | 0.055 | -0.089 | 0.147 |
| Asynchrony | DIRECT | **SD α richness** | **-0.599** | **0.007** | **0.104** | **-0.739** | **-0.259** |
|  |  | **CV tillage int.** | **0.243** | **-0.004** | **0.088** | **0.073** | **0.454** |
|  |  | **Mean pesticide use** | **0.198** | **-0.008** | **0.081** | **0.055** | **0.375** |
|  |  | **γ richness** | **0.226** | **-0.003** | **0.093** | **0.045** | **0.431** |
|  | INDIRECT | Mean prop. cropland | -0.079 | -0.003 | 0.043 | -0.160 | 0.003 |
|  |  | Mean tillage int. | 0.057 | -0.007 | 0.025 | 0.016 | 0.125 |
|  | TOTAL | Mean prop. cropland | -0.079 | -0.003 | 0.043 | -0.160 | 0.003 |
|  |  | Mean tillage int. | 0.057 | -0.007 | 0.025 | 0.016 | 0.125 |
|  |  | **SD α richness** | **-0.599** | **0.007** | **0.104** | **-0.739** | **-0.259** |
|  |  | **CV tillage int.** | **0.243** | **-0.004** | **0.088** | **0.073** | **0.454** |
|  |  | **Mean pesticide use** | **0.198** | **-0.008** | **0.081** | **0.055** | **0.375** |
|  |  | **γ richness** | **0.226** | **-0.003** | **0.093** | **0.045** | **0.431** |
|  | MED. | **γ richness** | -0.022 | -0.010 | 0.043 | -0.122 | 0.050 |
| Carabid | DIRECT | Mean crop pheno. homog**.** | 0.339 | -0.012 | 0.146 | -0.017 | 0.577 |
| biomass |  | **SD α richness** | **0.381** | **-0.010** | **0.105** | **0.192** | **0.615** |
| Mean |  | CV tillage int. | 0.232 | -0.020 | 0.112 | -0.007 | 0.445 |
| Value |  | **β-diversity** | **-0.197** | **-0.010** | **0.079** | **-0.333** | **-0.014** |
|  | INDIRECT | **Mean prop. cropland** | **-0.019** | **-0.003** | **0.016** | **-0.071** | **-0.001** |
|  |  | **Mean tillage int.** | **0.014** | **-0.001** | **0.010** | **0.004** | **0.049** |
|  |  | **Mean crop pheno. homog.** | **0.058** | **-0.001** | **0.028** | **0.009** | **0.146** |
|  |  | **SD α richness** | **-0.076** | **-0.001** | **0.035** | **-0.148** | **-0.011** |
|  |  | **γ richness** | **0.054** | **0.006** | **0.040** | **0.009** | **0.197** |
|  | TOTAL | **Mean prop. cropland** | **-0.019** | **-0.003** | **0.016** | **-0.071** | **-0.001** |
|  |  | **Mean tillage int.** | **0.014** | **-0.001** | **0.010** | **0.004** | **0.049** |
|  |  | **Mean crop pheno. homog.** | **0.397** | **-0.013** | **0.134** | **0.051** | **0.598** |
|  |  | **SD α richness** | **0.305** | **-0.010** | **0.108** | **0.110** | **0.511** |
|  |  | **CV tillage int.** | 0.232 | -0.020 | 0.112 | -0.007 | 0.445 |
|  |  | **γ richness** | **0.054** | **0.006** | **0.040** | **0.009** | **0.197** |
|  |  | **β-diversity** | **-0.197** | **-0.010** | **0.079** | **-0.333** | **-0.014** |
|  | MED. | γ richness | -0.005 | -0.004 | 0.015 | -0.052 | 0.010 |
|  |  | β-diversity | 0.031 | 0.000 | 0.036 | -0.044 | 0.104 |
| Carabid | DIRECT | **Mean crop pheno. homog.** | **-0.446** | **-0.002** | **0.083** | **-0.563** | **-0.250** |
| biomass |  | **Mean patch size** | **0.281** | **0.000** | **0.084** | **0.099** | **0.428** |
| stability |  | **β-diversity** | **-0.344** | **0.005** | **0.119** | **-0.551** | **-0.026** |
|  |  | **Asynchrony** | **0.202** | **-0.006** | **0.095** | **0.057** | **0.525** |
|  | INDIRECT | **Mean prop. cropland** | **-0.049** | **-0.001** | **0.028** | **-0.124** | **-0.007** |
|  |  | **Mean tillage int.** | **0.035** | **-0.002** | **0.021** | **0.012** | **0.090** |
|  |  | **Mean crop pheno. homog.** | **0.101** | **-0.007** | **0.047** | **0.009** | **0.193** |
|  |  | **SD α richness** | **-0.253** | **0.010** | **0.058** | **-0.421** | **-0.175** |
|  |  | **CV tillage int.** | **0.049** | **-0.004** | **0.026** | **0.010** | **0.142** |
|  |  | **Mean pesticide use** | **0.040** | **-0.003** | **0.024** | **0.006** | **0.093** |
|  |  | **γ richness** | **0.140** | **0.000** | **0.060** | **0.061** | **0.319** |
|  | TOTAL | **Mean prop. cropland** | **-0.049** | **-0.001** | **0.028** | **-0.124** | **-0.007** |
|  |  | **Mean tillage int.** | **0.035** | **-0.002** | **0.021** | **0.012** | **0.090** |
|  |  | **Mean crop pheno. homog.** | **-0.345** | **-0.008** | **0.075** | **-0.478** | **-0.178** |
|  |  | **SD α richness** | **-0.253** | **0.010** | **0.058** | **-0.421** | **-0.175** |
|  |  | **CV tillage int.** | **0.049** | **-0.004** | **0.026** | **0.010** | **0.142** |
|  |  | **Mean pesticide use** | **0.040** | **-0.003** | **0.024** | **0.006** | **0.093** |
|  |  | **Mean patch size** | **0.281** | **0.000** | **0.084** | **0.099** | **0.428** |
|  |  | **γ richness** | **0.140** | **0.000** | **0.060** | **0.061** | **0.319** |
|  |  | **β-diversity** | **-0.344** | **0.005** | **0.119** | **-0.551** | **-0.026** |
|  |  | **Asynchrony** | **0.202** | **-0.006** | **0.095** | **0.057** | **0.525** |
|  | MED. | γ richness | -0.014 | -0.004 | 0.028 | -0.086 | 0.036 |
|  |  | β-diversity | 0.054 | -0.001 | 0.060 | -0.052 | 0.188 |
|  |  | Asynchrony | 0.009 | -0.008 | 0.042 | -0.061 | 0.123 |

**SECTION B**

**Figure B Appendix 4**


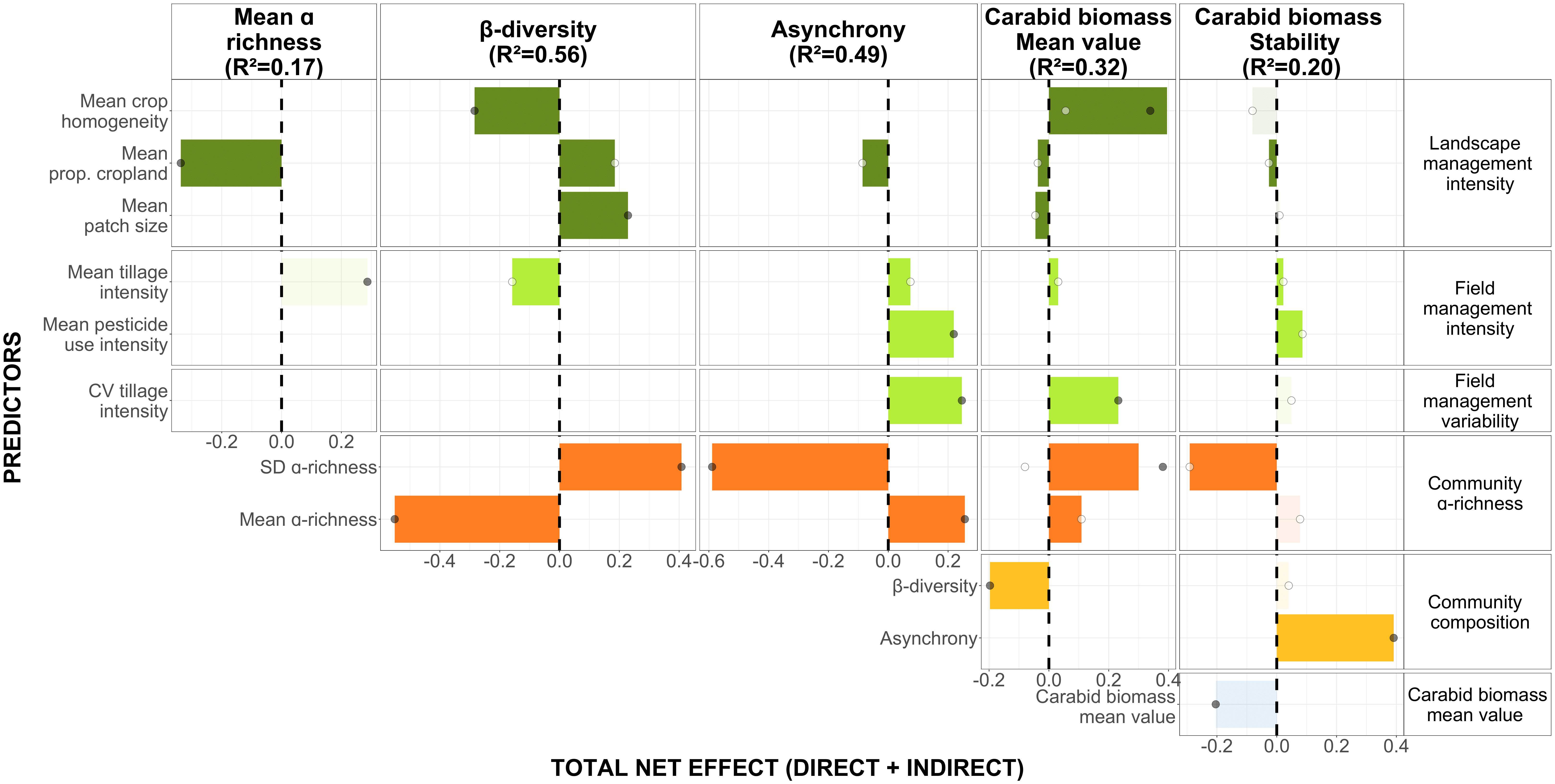


**Table B Appendix 4**

**Direct and indirect effects of the predictors based on the final version of the SEM.** Values were calculated using the semEff package (Murphy, 2022). Five models compose the SEM. Est, estimate; SE, standard error; LCI, lower confidence interval; UCI, upper confidence interval; MED, mediators. **This version includes carabid biomass stability like this CV-1 (σ/ μ ^-1^) instead of μ/σ in the version presented in the main text.**

| Response | Path | Predictor | Est. | Bias | SE | LCI | UCI |
| --- | --- | --- | --- | --- | --- | --- | --- |
| Mean α- | DIRECT | Mean tillage int. | 0.287 | 0.001 | 0.111 | -0.033 | 0.449 |
| richness |  | **Mean prop. cropland** | **-0.337** | **-0.001** | **0.113** | **-0.538** | **-0.105** |
|  | TOTAL | Mean tillage int. | 0.287 | 0.001 | 0.111 | -0.033 | 0.449 |
|  |  | **Mean prop. cropland** | **-0.337** | **-0.001** | **0.113** | **-0.538** | **-0.105** |
| β-diversity | DIRECT | **Mean patch size** | **0.229** | **-0.017** | **0.099** | **0.042** | **0.413** |
|  |  | **Mean crop pheno. homog.** | **-0.284** | **0.022** | **0.086** | **-0.495** | **-0.137** |
|  |  | **SD α richness** | **0.408** | **-0.011** | **0.082** | **0.260** | **0.568** |
|  |  | **Mean α richness** | **-0.551** | **0.025** | **0.091** | **-0.691** | **-0.329** |
|  | INDIRECT | Mean tillage int. | -0.158 | 0.009 | 0.060 | -0.280 | -0.028 |
|  |  | **Mean prop. cropland** | **0.185** | **-0.008** | **0.066** | **0.059** | **0.331** |
|  | TOTAL | **Mean tillage int.** | **-0.158** | **0.009** | **0.060** | **-0.280** | **-0.028** |
|  |  | **Mean prop. cropland** | **0.185** | **-0.008** | **0.066** | **0.059** | **0.331** |
|  |  | **Mean patch size** | **0.229** | **-0.017** | **0.099** | **0.042** | **0.413** |
|  |  | **Mean crop pheno. homog.** | **-0.284** | **0.022** | **0.086** | **-0.495** | **-0.137** |
|  |  | **SD α richness** | **0.408** | **-0.011** | **0.082** | **0.260** | **0.568** |
|  |  | **Mean α richness** | **-0.551** | **0.025** | **0.091** | **-0.691** | **-0.329** |
|  | MED. | Mean α richness | 0.027 | 0.000 | 0.091 | -0.095 | 0.278 |
| Asynchrony | DIRECT | **SD α richness** | **-0.589** | **0.011** | **0.100** | **-0.735** | **-0.319** |
|  |  | **CV tillage int.** | **0.246** | **-0.008** | **0.084** | **0.078** | **0.420** |
|  |  | **Mean pesticide use** | **0.219** | **-0.014** | **0.079** | **0.090** | **0.401** |
|  |  | **Mean α richness** | **0.256** | **-0.003** | **0.085** | **0.086** | **0.410** |
|  | INDIRECT | **Mean tillage int.** | **0.074** | **-0.004** | **0.034** | **0.033** | **0.176** |
|  |  | **Mean prop. cropland** | **-0.086** | **0.000** | **0.045** | **-0.216** | **-0.028** |
|  | TOTAL | **Mean tillage int.** | **0.074** | **-0.004** | **0.034** | **0.033** | **0.176** |
|  |  | **Mean prop. cropland** | **-0.086** | **0.000** | **0.045** | **-0.216** | **-0.028** |
|  |  | **SD α richness** | **-0.589** | **0.011** | **0.100** | **-0.735** | **-0.319** |
|  |  | **CV tillage int.** | **0.246** | **-0.008** | **0.084** | **0.078** | **0.420** |
|  |  | **Mean pesticide use** | **0.219** | **-0.014** | **0.079** | **0.090** | **0.401** |
|  |  | **Mean α richness** | **0.256** | **-0.003** | **0.085** | **0.086** | **0.410** |
|  | MED. | Mean α richness | -0.013 | -0.004 | 0.051 | -0.176 | 0.040 |
| Carabid | DIRECT | **Mean crop pheno. homog.** | **0.339** | **-0.027** | **0.123** | **0.098** | **0.544** |
| biomass |  | **SD α richness** | **0.381** | **0.005** | **0.083** | **0.179** | **0.549** |
| mean |  | **CV tillage int.** | **0.232** | **-0.024** | **0.101** | **0.010** | **0.386** |
| value |  | **β-diversity** | **-0.197** | **-0.012** | **0.077** | **-0.382** | **-0.080** |
|  | INDIRECT | **Mean tillage int.** | **0.031** | **-0.001** | **0.016** | **0.012** | **0.077** |
|  |  | **Mean prop. cropland** | **-0.037** | **-0.002** | **0.023** | **-0.114** | **-0.008** |
|  |  | **Mean patch size** | **-0.045** | **0.001** | **0.028** | **-0.133** | **-0.008** |
|  |  | **Mean crop pheno. homog.** | **0.056** | **-0.002** | **0.026** | **0.020** | **0.140** |
|  |  | **SD α richness** | **-0.080** | **-0.002** | **0.033** | **-0.167** | **-0.029** |
|  |  | **Mean α richness** | **0.109** | **0.002** | **0.048** | **0.045** | **0.233** |
|  | TOTAL | **Mean tillage int.** | **0.031** | **-0.001** | **0.016** | **0.012** | **0.077** |
|  |  | **Mean prop. cropland** | **-0.037** | **-0.002** | **0.023** | **-0.114** | **-0.008** |
|  |  | **Mean patch size** | **-0.045** | **0.001** | **0.028** | **-0.133** | **-0.008** |
|  |  | **Mean crop pheno. homog.** | **0.395** | **-0.029** | **0.111** | **0.176** | **0.600** |
|  |  | **SD α richness** | **0.300** | **0.004** | **0.077** | **0.094** | **0.430** |
|  |  | **CV tillage int.** | **0.232** | **-0.024** | **0.101** | **0.010** | **0.386** |
|  |  | **Mean α richness** | **0.109** | **0.002** | **0.048** | **0.045** | **0.233** |
|  |  | **β-diversity** | **-0.197** | **-0.012** | **0.077** | **-0.382** | **-0.080** |
|  | MED. | Mean α richness | -0.005 | -0.003 | 0.023 | -0.092 | 0.018 |
|  |  | β-diversity | 0.034 | -0.004 | 0.035 | -0.037 | 0.125 |
| Carabid | DIRECT | **Asynchrony** | **0.391** | **-0.011** | **0.105** | **0.176** | **0.589** |
| biomass |  | Carabid biomass mean value | -0.204 | -0.009 | 0.129 | -0.413 | 0.089 |
| Stability | INDIRECT | **Mean tillage int.** | **0.022** | **-0.002** | **0.017** | **0.001** | **0.083** |
|  |  | **Mean prop. cropland** | **-0.026** | **0.001** | **0.019** | **-0.092** | **-0.001** |
|  |  | Mean patch size | 0.009 | 0.000 | 0.010 | -0.002 | 0.040 |
|  |  | Mean crop pheno. homog. | -0.081 | 0.002 | 0.053 | -0.191 | 0.017 |
|  |  | **SD α richness** | **-0.291** | **0.011** | **0.084** | **-0.443** | **-0.129** |
|  |  | CV tillage int. | 0.049 | -0.005 | 0.060 | -0.083 | 0.163 |
|  |  | **Mean pesticide use** | **0.086** | **-0.006** | **0.042** | **0.032** | **0.194** |
|  |  | Mean α richness | 0.078 | -0.004 | 0.049 | -0.005 | 0.180 |
|  |  | β-diversity | 0.040 | 0.002 | 0.031 | -0.014 | 0.128 |
|  | TOTAL | **Mean tillage int.** | **0.022** | **-0.002** | **0.017** | **0.001** | **0.083** |
|  |  | **Mean prop. cropland** | **-0.026** | **0.001** | **0.019** | **-0.092** | **-0.001** |
|  |  | Mean patch size | 0.009 | 0.000 | 0.010 | -0.002 | 0.040 |
|  |  | Mean crop pheno. homog. | -0.081 | 0.002 | 0.053 | -0.191 | 0.017 |
|  |  | **SD α richness** | **-0.291** | **0.011** | **0.084** | **-0.443** | **-0.129** |
|  |  | CV tillage int. | 0.049 | -0.005 | 0.060 | -0.083 | 0.163 |
|  |  | **Mean pesticide use** | **0.086** | **-0.006** | **0.042** | **0.032** | **0.194** |
|  |  | Mean α richness | 0.078 | -0.004 | 0.049 | -0.005 | 0.180 |
|  |  | β-diversity | 0.040 | 0.002 | 0.031 | -0.014 | 0.128 |
|  |  | **Asynchrony** | **0.391** | **-0.011** | **0.105** | **0.176** | **0.589** |
|  |  | Carabid biomass mean value | -0.204 | -0.009 | 0.129 | -0.413 | 0.089 |
|  | MED. | Mean α richness | -0.004 | 0.000 | 0.013 | -0.046 | 0.012 |
|  |  | β-diversity | -0.007 | 0.001 | 0.009 | -0.050 | 0.002 |
|  |  | Asynchrony | 0.047 | -0.006 | 0.094 | -0.127 | 0.297 |
|  |  | Carabid biomass mean value | -0.161 | 0.006 | 0.106 | -0.381 | 0.037 |

**SECTION C**

**Figure C Appendix 4**


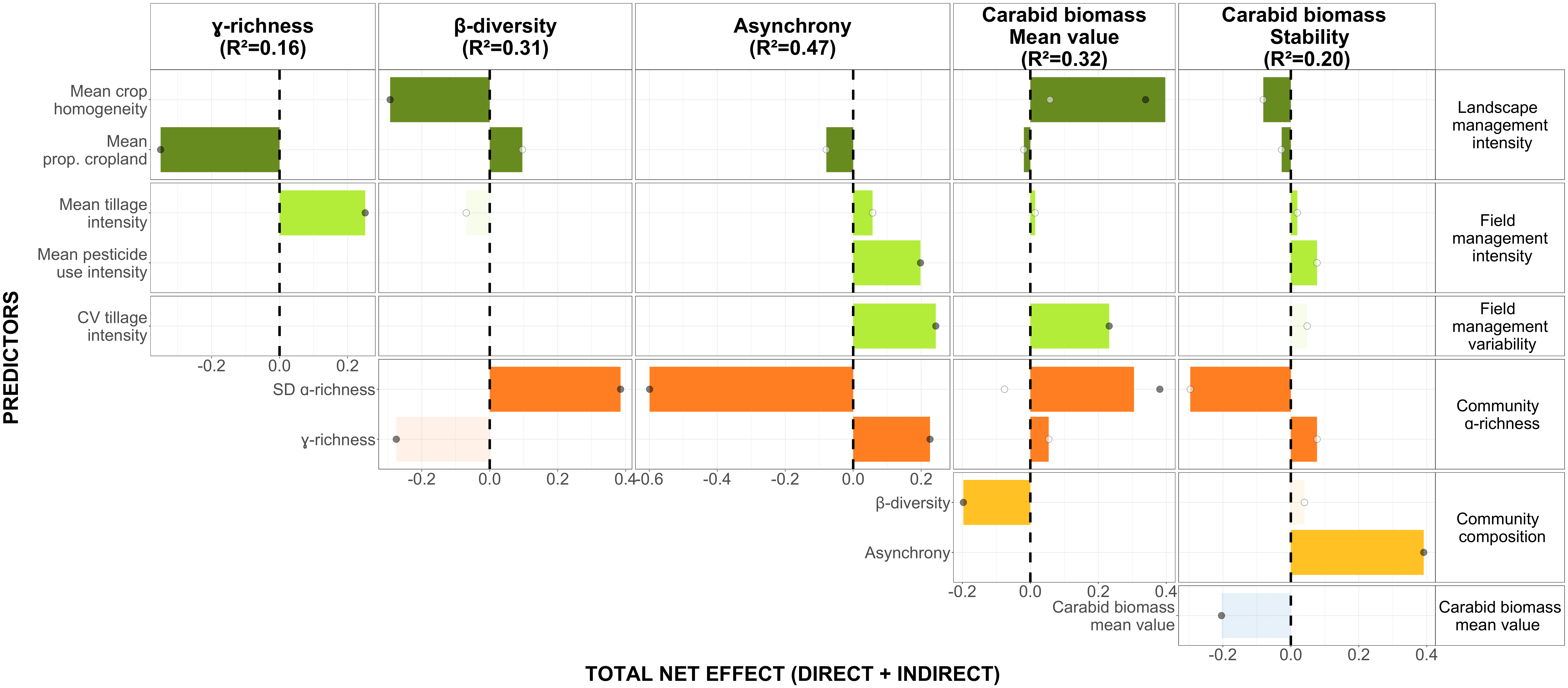


**Table C Appendix 4**

**Direct and indirect effects of the predictors based on the final version of the SEM.** Values were calculated using the semEff package (Murphy, 2022). Four models remain in the final version of the partial SEM. Est, estimate; SE, standard error; LCI, lower confidence interval; UCI, upper confidence interval; MED, mediators. **This version includes 1) ɣ-richness instead of mean α-richness and 2) CV-1 (σ/ μ -1) instead of μ/σ in the version presented in the main text.**

| Response | Path | Predictor | Estimates | Bias | SE | LCI | UCI |
| --- | --- | --- | --- | --- | --- | --- | --- |
| γ-richness | DIRECT | **Mean prop. cropland** | **-0.350** | **0.002** | **0.109** | **-0.523** | **-0.080** |
|  |  | **Mean tillage int.** | **0.252** | **0.022** | **0.099** | **0.063** | **0.447** |
|  | TOTAL | **Mean prop. cropland** | **-0.350** | **0.002** | **0.109** | **-0.523** | **-0.080** |
|  |  | **Mean tillage int** | **0.252** | **0.022** | **0.099** | **0.063** | **0.447** |
| β-diversity | DIRECT | **Mean crop pheno. homog.** | **-0.293** | **0.020** | **0.107** | **-0.543** | **-0.074** |
|  |  | **SD α richness** | **0.385** | **-0.002** | **0.110** | **0.065** | **0.538** |
|  |  | γ richness | -0.275 | 0.007 | 0.109 | -0.434 | 0.039 |
|  | INDIRECT | **Mean prop. cropland** | **0.096** | **0.000** | **0.054** | **0.010** | **0.241** |
|  |  | Mean tillage int. | -0.069 | -0.002 | 0.036 | -0.147 | 0.002 |
|  | TOTAL | **Mean prop. cropland** | **0.096** | **0.000** | **0.054** | **0.010** | **0.241** |
|  |  | Mean tillage int. | -0.069 | -0.002 | 0.036 | -0.147 | 0.002 |
|  |  | **Mean crop pheno. homog.** | **-0.293** | **0.020** | **0.107** | **-0.543** | **-0.074** |
|  |  | **SD α richness** | **0.385** | **-0.002** | **0.110** | **0.065** | **0.538** |
|  |  | γ richness | -0.275 | 0.007 | 0.109 | -0.434 | 0.039 |
|  | MED. | γ richness | 0.027 | -0.002 | 0.048 | -0.043 | 0.223 |
| Asynchrony | DIRECT | **SD α richness** | **-0.599** | **0.002** | **0.103** | **-0.732** | **-0.301** |
|  |  | **CV tillage int.** | **0.243** | **-0.003** | **0.083** | **0.044** | **0.390** |
|  |  | **Mean pesticide use** | **0.198** | **-0.019** | **0.078** | **0.024** | **0.333** |
|  |  | **γ richness** | **0.226** | **-0.008** | **0.096** | **0.073** | **0.484** |
|  | INDIRECT | **Mean prop. cropland** | **-0.079** | **0.001** | **0.047** | **-0.219** | **-0.011** |
|  |  | **Mean tillage int.** | **0.057** | **-0.002** | **0.026** | **0.016** | **0.136** |
|  | TOTAL | **Mean prop. cropland** | **-0.079** | **0.001** | **0.047** | **-0.219** | **-0.011** |
|  |  | **Mean tillage int.** | **0.057** | **-0.002** | **0.026** | **0.016** | **0.136** |
|  |  | **SD α richness** | **-0.599** | **0.002** | **0.103** | **-0.732** | **-0.301** |
|  |  | **CV tillage int.** | **0.243** | **-0.003** | **0.083** | **0.044** | **0.390** |
|  |  | **Mean pesticide use** | **0.198** | **-0.019** | **0.078** | **0.024** | **0.333** |
|  |  | **γ richness** | **0.226** | **-0.008** | **0.096** | **0.073** | **0.484** |
|  | MED. | γ richness | -0.022 | -0.001 | 0.042 | -0.149 | 0.036 |
| Carabid | DIRECT | **Mean crop pheno. homog.** | **0.339** | **-0.041** | **0.145** | **0.026** | **0.605** |
| biomass |  | **SD α richness** | **0.381** | **-0.002** | **0.095** | **0.188** | **0.600** |
| mean |  | **CV tillage int.** | **0.232** | **-0.043** | **0.109** | **0.046** | **0.421** |
| value |  | **β-diversity** | **-0.197** | **-0.010** | **0.070** | **-0.336** | **-0.049** |
|  | INDIRECT | **Mean prop. cropland** | **-0.019** | **-0.002** | **0.016** | **-0.085** | **-0.002** |
|  |  | **Mean tillage int.** | **0.014** | **0.001** | **0.010** | **0.002** | **0.050** |
|  |  | **Mean crop pheno. homog.** | **0.058** | **-0.003** | **0.026** | **0.015** | **0.137** |
|  |  | **SD α richness** | **-0.076** | **-0.003** | **0.035** | **-0.150** | **-0.015** |
|  |  | **γ richness** | **0.054** | **0.003** | **0.035** | **0.011** | **0.183** |
|  | TOTAL | **Mean prop. cropland** | **-0.019** | **-0.002** | **0.016** | **-0.085** | **-0.002** |
|  |  | **Mean tillage int.** | **0.014** | **0.001** | **0.010** | **0.002** | **0.050** |
|  |  | **Mean crop pheno. homog.** | **0.397** | **-0.044** | **0.139** | **0.135** | **0.640** |
|  |  | **SD α richness** | **0.305** | **-0.006** | **0.098** | **0.092** | **0.521** |
|  |  | **CV tillage int.** | **0.232** | **-0.043** | **0.109** | **0.046** | **0.421** |
|  |  | **γ richness** | **0.054** | **0.003** | **0.035** | **0.011** | **0.183** |
|  |  | **β-diversity** | **-0.197** | **-0.010** | **0.070** | **-0.336** | **-0.049** |
|  | MED. | γ richness | -0.005 | -0.001 | 0.013 | -0.083 | 0.008 |
|  |  | β-diversity | 0.031 | -0.005 | 0.035 | -0.030 | 0.133 |
| Carabid | DIRECT | **Asynchrony** | **0.391** | **-0.002** | **0.096** | **0.166** | **0.536** |
| biomass |  | Carabid biomass mean value | -0.204 | -0.011 | 0.117 | -0.447 | 0.040 |
| stability | INDIRECT | **Mean prop. cropland** | **-0.027** | **0.001** | **0.019** | **-0.103** | **-0.005** |
|  |  | **Mean tillage int.** | **0.019** | **-0.001** | **0.012** | **0.004** | **0.060** |
|  |  | **Mean crop pheno. homog.** | **-0.081** | **0.007** | **0.048** | **-0.197** | **-0.005** |
|  |  | **SD α richness** | **-0.296** | **0.003** | **0.077** | **-0.451** | **-0.132** |
|  |  | CV tillage int. | 0.048 | 0.003 | 0.049 | -0.086 | 0.118 |
|  |  | **Mean pesticide use** | **0.077** | **-0.006** | **0.038** | **0.019** | **0.161** |
|  |  | **γ richness** | **0.077** | **-0.005** | **0.042** | **0.016** | **0.189** |
|  |  | β-diversity | 0.040 | 0.003 | 0.026 | -0.010 | 0.104 |
|  | TOTAL | **Mean prop. cropland** | **-0.027** | **0.001** | **0.019** | **-0.103** | **-0.005** |
|  |  | **Mean tillage int.** | **0.019** | **-0.001** | **0.012** | **0.004** | **0.060** |
|  |  | **Mean crop pheno. homog.** | **-0.081** | **0.007** | **0.048** | **-0.197** | **-0.005** |
|  |  | **SD α richness** | **-0.296** | **0.003** | **0.077** | **-0.451** | **-0.132** |
|  |  | CV tillage int. | 0.048 | 0.003 | 0.049 | -0.086 | 0.118 |
|  |  | **Mean pesticide use** | **0.077** | **-0.006** | **0.038** | **0.019** | **0.161** |
|  |  | **γ richness** | **0.077** | **-0.005** | **0.042** | **0.016** | **0.189** |
|  |  | β-diversity | 0.040 | 0.003 | 0.026 | -0.010 | 0.104 |
|  |  | **Asynchrony** | **0.391** | **-0.002** | **0.096** | **0.166** | **0.536** |
|  |  | Carabid biomass mean value | -0.204 | -0.011 | 0.117 | -0.447 | 0.040 |
|  | MED. | γ richness | -0.008 | 0.001 | 0.015 | -0.061 | 0.010 |
|  |  | β-diversity | -0.006 | 0.001 | 0.009 | -0.045 | 0.003 |
|  |  | Asynchrony | 0.018 | -0.012 | 0.075 | -0.136 | 0.191 |
|  |  | **Carabid biomass mean value** | **-0.160** | **0.016** | **0.098** | **-0.374** | **-0.033** |
